# A simple, robust, universal assay for real-time enzyme monitoring by signalling changes in nucleoside phosphate anion concentration using a europium(iii)-based anion receptor†
†Electronic supplementary information (ESI) available. See DOI: 10.1039/c9sc01552c


**DOI:** 10.1039/c9sc01552c

**Published:** 2019-05-01

**Authors:** Sarah H. Hewitt, Rozee Ali, Romain Mailhot, Chloe R. Antonen, Charlotte A. Dodson, Stephen J. Butler

**Affiliations:** a Department of Chemistry , Loughborough University , Epinal Way , Loughborough , LE11 3TU , UK . Email: S.J.Butler@lboro.ac.uk; b Department of Pharmacy & Pharmacology , University of Bath , Claverton Down , Bath , BA2 7AY , UK

## Abstract

Enzymes that consume and produce nucleoside polyphosphate (NPP) anions represent major targets in drug discovery. For example, protein kinases are one of the largest classes of drug targets in the fight against cancer. The accurate determination of enzyme kinetics and mechanisms is a critical aspect of drug discovery research. To increase confidence in the selection of lead drug compounds it is crucial that pharmaceutical researchers have robust, affordable assays to measure enzyme activity accurately. We present a simple, sensitive microplate assay for real-time monitoring of a range of pharmaceutically important enzyme reactions that generate NPP anions, including kinases and glycosyltransferases. Our assay utilises a single, stable europium(iii) complex that binds reversibly to NPP anions, signalling the dynamic changes in NPP product/substrate ratio during an enzyme reaction using time-resolved luminescence. This supramolecular approach to enzyme monitoring overcomes significant limitations in existing assays, obviating the need for expensive antibodies or equipment, chemically labelled substrates or products and isolation or purification steps. Our label and antibody-free method enables rapid and quantitative analysis of enzyme activities and inhibition, offering a potentially powerful tool for use in drug discovery, suitable for high-throughput screening of inhibitors and accurate measurements of enzyme kinetic parameters.

## Abstract 2

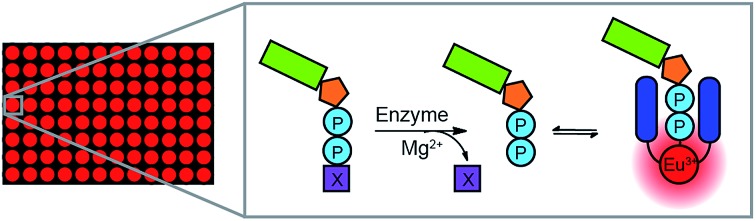
A simple, sensitive microplate assay for real-time enzyme monitoring, using a lanthanide-based anion receptor, could increase productivity in the drug discovery pipeline.

## Introduction

Monitoring enzymatic activity is of fundamental importance to industrial and biomedical researchers. It enables enzyme kinetics and thus mechanisms to be determined; a critical first step in the discovery of potent enzyme inhibitors and activators.^1^ Nucleoside polyphosphate (NPP) anions are involved in a number of pharmaceutically important enzyme reactions including kinases, GTPases and glycosyltransferases (GTs). Protein kinases catalyse the phosphorylation of amino acid residues (*e.g.* Ser, Thr, Tyr) on proteins, converting ATP into ADP in the process. Misregulation of kinase activity is a primary cause of many types of cancer; therefore, protein kinases are a major target for oncology drug discovery.^2^ GTs catalyse the transfer of a sugar from a donor (*e.g.* UDP-sugar) to a variety of acceptors (*e.g.* oligosaccharides, proteins, lipids)^3^ and have been identified as promising drug targets to treat tuberculosis and metabolic disorders; yet only two GT inhibitors are in clinical use.^4^ Progress in GT inhibitor development is limited by the lack of robust, label-free tools for conducting high-throughput screening (HTS) assays. Currently, there is no low-cost method available for real-time monitoring of kinase or GT reactions,^4^ limiting progress in understanding their mechanisms and inhibition, increasing the risk of late-stage clinical failure of drug candidates.

The majority of commercial enzyme assays (ADPGlo™, HTRF®, DELFIA® for kinases, UDPGlo™ for GTs), are restricted to single end-point measurements, making the accurate determination of kinetic parameters (*K*_m_, *k*_cat_) difficult and time consuming, particularly for enzymes with complicated mechanisms. Many enzyme assays require expensive antibodies to detect a specific product and require isolation and washing steps preventing real-time measurements.^5^ Other assays remove the need for antibodies but require substrates with luminescent or radioactive labels.^6^–^8^ For example, peptide-sensor conjugates have been used to probe kinase activity, involving chemical modification of a peptide substrate with phosphate recognition groups, which signal phosphorylation usually by a FRET interaction.^9^ However, this approach precludes the use of natural substrates, increasing uncertainty in ‘hit’ compounds. The high cost of these reagents and time required to validate the assays places a strain on drug development. To increase productivity earlier in the drug discovery process, a low-cost, label and antibody-free method for real-time reporting of enzyme activity is needed.

Synthetic host molecules that bind selectively to a target analyte offer an attractive alternative approach for monitoring enzyme reactions. Cucurbituril and calixarene hosts have been employed successfully in supramolecular enzyme assays.^10^,^11^ Cucurbituril hosts bind ammonium ions selectively, displacing a fluorescent dye from the host, allowing real-time analysis of methyltransferase and demethylase reactions.^12^ However, cucurbituril hosts do not bind to phosphate derivatives and therefore cannot be used for monitoring enzymes such as kinases, GTPases or GTs. Kinase activity has been monitoring by using cavitand/fluorophore pairs to discriminate between phosphopeptides in an array-based format.^13^,^14^ An alternative approach is to use a single receptor that can discriminate between NPP anions, whilst showing negligible binding to phosphorylated peptides. This could provide a universal assay platform for real-time monitoring of NPP-anion utilising enzyme reactions.

The creation of receptors that can discriminate between NPP anions (*e.g.* ATP, ADP, GTP, UDP) is particularly challenging due to similarities in anion structure, size and charge.^15^ Hence, examples of NPP-selective receptors are quite rare; the majority utilise positively charged recognition groups to engage in electrostatic interactions with the phosphate groups of NPPs. Dinuclear Zn(ii) complexes have shown high affinity for NPPs, pyrophosphate and phosphorylated peptides,^16^,^17^ and have been used for both end-point and real-time monitoring of enzyme reactions.^18^,^19^ However, such receptors exhibit similar affinities for tri- and diphosphate anions, and often induce similar fluorescence responses. Additionally, the fluorescence is short-lived and can be difficult to distinguish from the background fluorescence of biological substrates, decreasing the signal to noise ratio.

Receptors based on luminescent lanthanide(III) complexes^20^,^21^ offer unique photophysical properties that are especially useful in enzyme assays,^6^,^22^,^23^ including well-defined emission spectral bands that allow ratiometric measurements (enhancing signal to noise) and long luminescence lifetimes that enable time-resolved measurements, eliminating background autofluorescence from biological assay components. A number of emissive Eu(iii) and Tb(iii) receptors have been designed to bind small anions (*e.g.* HCO_3_^–^,^24^ F^–^,^25^,^26^ CN^–^27).^28^,^29^ However, probes capable of sensing larger NPP anions are quite rare and usually act as ‘on-off’ probes, where anion binding causes quenching of luminescence by displacing the sensitizing ‘antenna’, or by energy transfer to the nucleotide base.^30^,^31^ Notably, Pierre developed an ATP-selective Tb(iii) complex, wherein π–π stacking of the adenosine base and phenanthridine antenna causes luminescence quenching.^32^,^33^

**Fig. 1:**
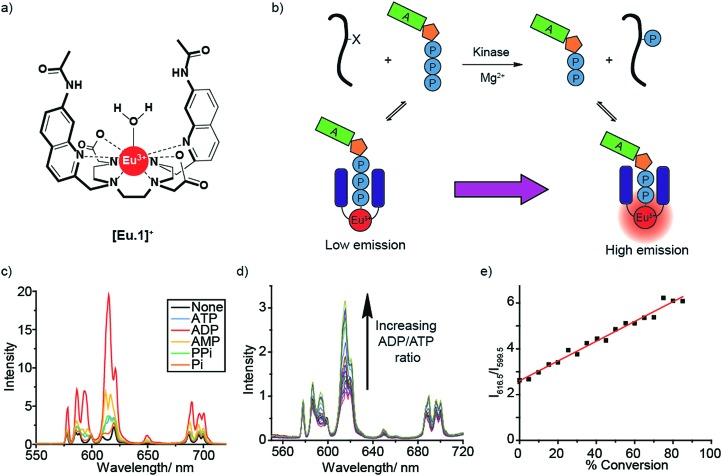
(a) Structure of Eu(iii)-based phosphoanion receptor **[Eu.1]^+^**. (b) Cartoon illustrating real-time monitoring of the kinase-catalyzed conversion of ATP to ADP using **[Eu.1]^+^**. (c) Emission spectra of **[Eu.1]^+^** (8 μM) in the presence of different phosphoanions (1 mM) and MgCl_2_ (5 mM) in 10 mM HEPES, pH 7.0, *λ*_exc_ = 330 nm. (d) and (e) Titration of ADP (1 mM) into ATP (1 mM) in the presence of **[Eu.1]^+^** (8 μM) and MgCl_2_ (5 mM) showing the change in emission spectra (d) and the change in the emission intensity ratio at 616.5/599.5 nm as the mole fraction of ADP increases (e) in 10 mM HEPES, pH 7.0, *λ*_exc_ = 330 nm.

We recently reported a stable cationic Eu(iii) complex **[Eu.1]^+^** (Fig. 1a), which binds reversibly to ATP, ADP and AMP,^34^ displacing the coordinated water molecule and giving rise to significantly different emission spectra, particularly in the presence of 3 mM Mg^2+^ ions, a critical cofactor for enzymes utilising NPP anions. We used this to develop a fluorimeter-based assay to monitor a kinase reaction in real-time. The Eu(iii) probe showed a linear increase in the ratiometric emission at 616.5/599.5 nm against the ADP/ATP ratio, allowing the luminescence signal to be directly correlated to the progress of a kinase catalysed phosphorylation reaction.

Here, we report the complete development of our Eu(iii) probe into a miniaturised assay for real-time monitoring of a wide-range of NPP-utilising enzyme reactions, including kinases, glycosyltransferases and phosphodiesterases. We have utilised the long luminescence lifetime of **[Eu.1]^+^** to record time-resolved measurements of the NPP substrate/product ratio using a standard microplate reader, thereby enhancing signal-to-noise by removing background autofluorescence from the sample. We demonstrate the robustness of our assay to: (1) monitor multiple enzyme reactions at once, in a wide range of biologically relevant conditions; (2) derive accurate kinetic parameters allowing quantitative analysis of enzyme inhibitors; (3) monitor multistep enzymatic processes in one-pot. Our single Eu(iii) probe has significant advantages over existing enzyme assays, enabling real-time analysis of reaction kinetics whilst eliminating the need for expensive antibodies, labelled substrates, or isolation/purification steps.

## Results and discussion

Molecular host **[Eu.1]^+^** possesses key design features for the discrimination of NPP anions under physiological conditions. **[Eu.1]^+^** is a stable, cationic Eu(iii) complex that is synthesised in a modular fashion,^26^ and is able to bind reversibly to ATP and ADP, by means of a phosphate–Eu(iii) interaction, strengthened by hydrogen bonding to the quinoline amide arms.^34^ Addition of 1 mM ATP and ADP to **[Eu.1]^+^** gives rise to distinctly different emission spectra at pH 7.0 (Fig. S1†); the overall emission intensity of **[Eu.1]^+^** increases due to displacement of the coordinated, quenching water molecule by the phosphoanion, with a particular increase in emission of the hypersensitive Δ*J* = 2 band (605–630 nm). Addition of ADP causes a larger increase in emission intensity compared to ATP, with the presence of 5 mM Mg^2+^ ions amplifying this difference (Fig. 1c). This is attributed to the high affinity of **[Eu.1]^+^** for ADP in aqueous buffer (10 mM HEPES, pH 7.0, log *K*_a_ = 4.6) and the higher competitive binding of Mg^2+^ to ATP over ADP. The use of physiological levels of anions (1 mM)^35^ and Mg^2+^ ions is especially important for real-world applications since ATP is present at millimolar concentrations *in vivo* and exists predominantly as ATP-Mg^2-^,^36^ and Mg^2+^ ions act as cofactors for most enzymes that utilise NPP substrates.^37^,^38^

A significant advantage of **[Eu.1]^+^** is its high discriminatory behaviour between ATP and ADP in the presence of Mg^2+^ ions, since several host–guest interactions that operate in water dissociate upon adding biologically relevant amounts of cations or salt.^11^ Crucially, it allows direct monitoring of the change in the ADP/ATP ratio. On titration of ADP into ATP in the presence of **[Eu.1]^+^** and Mg^2+^ (Fig. 1d), the emission intensity ratio increases linearly (Fig. 1e), as the **[Eu.1]**–ATP host–guest complex is competitively replaced by the **[Eu.1]**-ADP complex. This experiment simulates a kinase reaction, where ATP is gradually converted to ADP, with a concomitant phosphorylation of the peptide substrate. The linear increase in emission intensity of **[Eu.1]^+^** with ADP/ATP ratio means the emission can be directly correlated to the progress of a kinase reaction in real-time.

### Signalling the ADP/ATP ratio in microplate format using time-resolved luminescence

To develop **[Eu.1]^+^** into a practical biological assay, it is necessary to convert our cuvette-based experiment to a high-throughput microplate assay, enabling multiple enzyme reactions to be monitored simultaneously. To this end, we added differing ratios of ATP and ADP (total 1 mM) to a 384-well plate in the presence of **[Eu.1]^+^** (8 μM, pH 7.0, 10 mM HEPES, 5 mM MgCl_2_), simulating a kinase reaction. As expected, the emission intensity of the Δ*J* = 2 band (605–630 nm) increased linearly with the increasing ratio of ADP/ATP (Fig. S2a†).

**Fig. 2:**
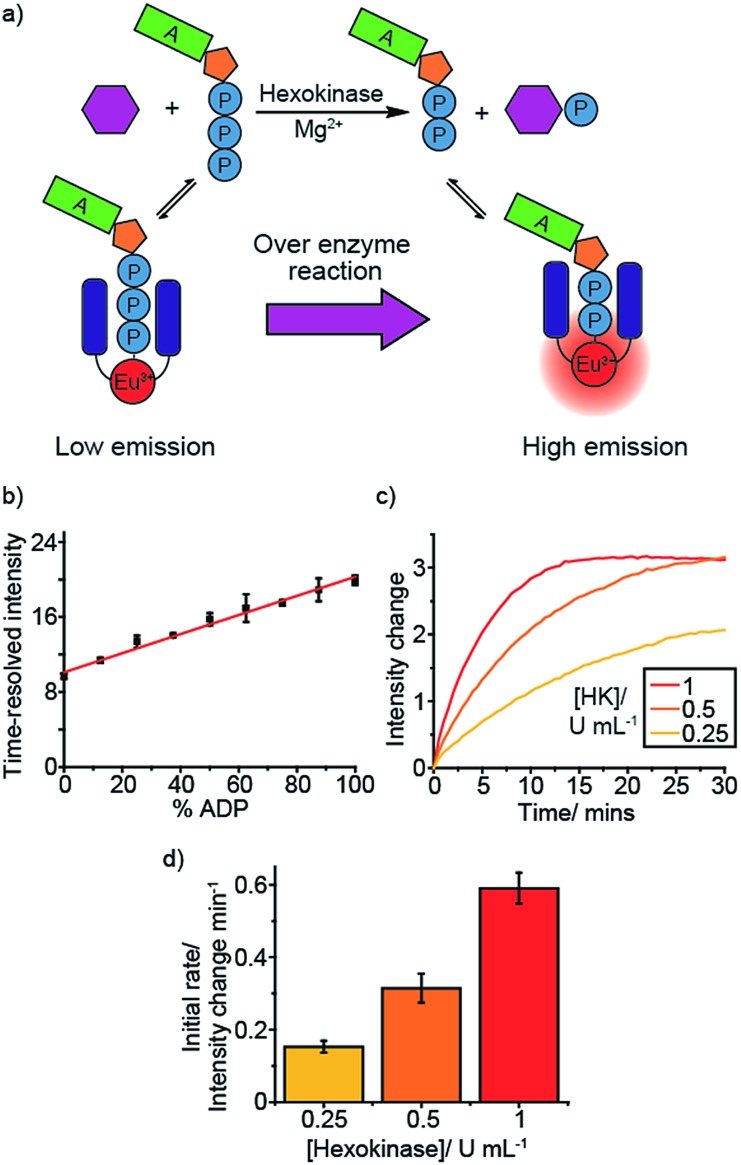
Microplate-based real-time monitoring of a kinase reaction. (a) Cartoon depicting the use of **[Eu.1]^+^** to monitor hexokinase. (b) Kinase simulation in standard assay conditions (1 mM ATP + ADP, 5 mM MgCl_2_, 8 μM **[Eu.1]^+^**, 10 mM HEPES, pH 7.0), measuring the time-resolved luminescence intensity (*λ*_exc_ = 292–366 nm, *λ*_em_ = 615–625 nm, integration time = 60–400 μs) of differing ratios of ADP/(ATP + ADP) (% ADP). (c) and (d) Real-time monitoring of different concentrations of hexokinase using the time-resolved luminescence intensity of **[Eu.1]^+^** and calculation of initial rates (d). Conditions: 1 mM ATP, 5 mM MgCl_2_, 10 mM glucose, 8 μM **[Eu.1]^+^**, 10 mM HEPES, pH 7.0, *λ*_exc_ = 292–366 nm, *λ*_em_ = 615–625 nm, integration time = 60–400 μs.

Next, we utilised the long-lived emission of **[Eu.1]^+^** (0.5 milliseconds in water)^34^ to record time-resolved luminescence measurements of the Δ*J* = 2 band (integration time = 60–400 μs), gating out short-lived fluorescence from biological fluorophores (*e.g.* tryptophan and tyrosine residues on proteins). Crucially, a time-resolved measurement gave a linear emission response to the ADP/ATP ratio (Fig. 2b) with significantly increased signal to noise, allowing the luminescence signal to be correlated to the course of a kinase reaction. To confirm this, we used hexokinase as a model enzyme. Hexokinase is a well-studied, commercially available metabolic kinase that catalyses the phosphorylation of glucose in the first step of glycolysis (Fig. 2a), converting ATP to ADP in the process (Fig. 2a). By following the increase in the time-resolved Δ*J* = 2 emission intensity (Fig. 2c) of **[Eu.1]^+^**, the enzyme reaction was monitored as a function of time as the ADP/ATP ratio increases, confirming our ability to monitor kinase reactions in a microplate format. Decreasing the concentration of hexokinase by a factor of two resulted in a decrease in the initial rate of luminescence intensity increase by the same order (Fig. 2d).

To verify that **[Eu.1]^+^** does not perturb the enzyme reaction rate, we monitored the hexokinase reaction at different concentrations of **[Eu.1]^+^** (4–64 μM), in otherwise identical conditions. This showed the same percentage changes between the maximum and minimum intensities (corresponding to the maximum and minimum percentage of ADP) at the same time points (Fig. S3†), confirming that the presence of **[Eu.1]^+^** has very little impact on the enzyme reaction rate.

If **[Eu.1]^+^** is to be used in a biological context it must be able to tolerate different mixtures of substrates, additives, salts and variations in pH. We tested the robustness of our assay by evaluating the ability of **[Eu.1]^+^** to report hexokinase activity in a variety of biologically relevant conditions (Table 1, Fig. S3–S22†), including different additives (*e.g.* DMSO, BSA), temperatures, pHs, buffers, ionic strengths, concentrations of MgCl_2_, substrates (other hexoses), concentrations of ATP and metal cofactor (Mg^2+^*vs.* Ca^2+^). Gratifyingly, it was possible to simulate and/or monitor a hexokinase reaction using **[Eu.1]^+^** in a wide range of conditions (Fig. S3–S22†).

**Table 1:** Different assay conditions tested for kinase reaction monitoring, assessed using kinase simulations^*a*^ and confirmed by real-time hexokinase monitoring in selected cases^*b*^

Condition	Range	Figures^*c*^
[ATP]	0.1–5.0 mM	S4–S6
[MgCl_2_]	0–20 mM	S7–S9
Ionic strength	0–500 mM NaCl	S10
Buffer	HEPES (10 mM, pH 7.0), tris (10, 20, 50, and 100 mM, pH 7.5)	S11
Substrates	Glucose, fructose, mannose	S12
**[Eu.1]^+^**	0.125–64 μM	S3, S13–S14
pH (in 50 mM tris)	7.0–8.5	S15
Buffer additives	0.1 mg mL^–1^ BSA, 0.02% Triton X-100, 10% DMSO, 10% glycerol, 200 mM NaCl, 200 mM KCl	S16–S18
M^2+^	MgCl_2_, CaCl_2_	S19–S20
Temperature	20–45 °C	S21–S22

^*a*^Conditions (unless stated otherwise): 1 mM ATP + ADP, 5 mM MgCl_2_, 8 μM **[Eu.1]^+^**, 10 mM HEPES, pH 7.0, *λ*_exc_ = 292–366 nm, *λ*_em_ = 615–625 nm, integration time = 60–400 μs.

^*b*^Conditions: Identical to kinase simulation conditions, except for 1 mM ATP (no ADP), 10 mM glucose.

^*c*^See ESI.

Importantly, the kinase reaction could be performed over a wide ATP concentration range (0.1–5 mM), including physiological ATP levels, and is thus suitable for monitoring low activity kinases, an area of increasing pharmaceutical interest but where existing assays offer very little.^39^ It is also worth noting that the concentration of **[Eu.1]^+^** can be tuned down to the sub-micromolar range (0.125 μM), ensuring that very small amounts of ATP or ADP are sequestered at any given time during the reaction. Thus, our supramolecular assay can be tailored readily to the conditions appropriate for the kinase of interest, a critical factor when considering a new tool for high-throughput screening applications.

**Fig. 3:**
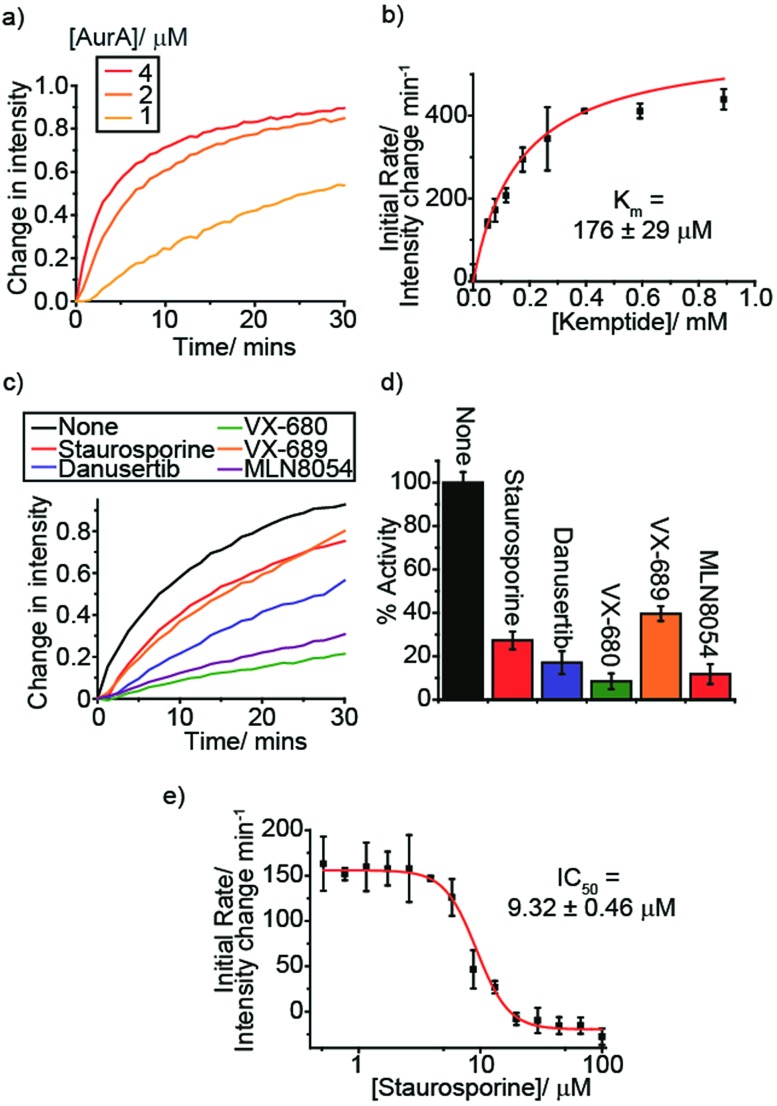
Real-time monitoring of Aurora A kinase reactions. (a) Enzyme reaction at different concentrations of Aurora A. (b) Initial AurA kinase (1 μM) reaction rate at different concentrations of the peptide substrate, kemptide, fitted to a Michaelis–Menten equation. (c) and (d) Inhibition of AurA (1 μM) by a range of known inhibitors measured in real-time (c), each showing a decrease in initial reaction rate (d, shown as %activity of the enzyme without inhibitor). (e) Titration of staurosporine into Aurora A (50 nM) reaction to derive an IC_50_. Conditions: 1 mM ATP, 0.5 mM kemptide (except kemptide titration), 0.25 mM DTT, 5 mM MgCl_2_, 8 μM **[Eu.1]^+^**, 2.5% glycerol, 50 mM NaCl, 10 mM HEPES, pH 7.0, *λ*_exc_ = 292–366 nm, *λ*_em_ = 615–625 nm, integration time = 60–400 μs), 2.5% DMSO for inhibitor screen, 5% DMSO for staurosporine titration.

To demonstrate the versatility of **[Eu.1]^+^** we selected a therapeutically targetable protein kinase, Aurora A kinase (AurA), and monitored this enzyme in more complex buffer conditions, utilising a peptide substrate. AurA regulates entry into mitosis and other events integral to cell proliferation,^40^ and is a target of several cancer drug discovery programmes.^41^ Initially, we monitored the AurA reaction at different enzyme concentrations (Fig. 3a). As AurA catalyses the phosphorylation of the substrate, kemptide (LRRASLG), there is an increase in the time-resolved Δ*J* = 2 emission intensity of **[Eu.1]^+^**, as observed in the model hexokinase reaction. Increasing the AurA concentration lead to a faster emission intensity increase, consistent with an increase in reaction rate.

### Monitoring kinase inhibition and determining kinetic parameters

An essential feature of enzyme assays is the ability to derive accurate kinetic parameters, such as substrate *K*_m_ values and inhibition data (IC_50_/*K*_i_ values). This is key to understanding enzyme mechanisms and to inform the development of new inhibitors. With this in mind, we varied the substrate concentration for both hexokinase and Aurora A (fructose and kemptide, respectively). For the different substrate concentrations, the initial rate was calculated as the change in intensity per minute over the first ∼25% of the reaction, fitting the data to a Michaelis–Menten equation (Fig. S23† and 3b). This data allowed substrate *K*_m_ values to be determined, giving 252 ± 43 μM for fructose/hexokinase and 176 ± 29 μM for kemptide/AurA. The calculated values are comparable to the literature (330 μM for each substrate).^42^–^44^

Next, we evaluated the ability of our probe to perform quantitative analysis of inhibition. First, we added various known AurA inhibitors and, in all cases, observed a decrease in the enzyme reaction rate compared to in the absence of inhibitor (Fig. 3c and d). Addition of TPX2, an Aurora A activator, lead to an increase in the enzyme reaction rate (Fig. S24†), as expected. The effect of one of the inhibitors, staurosporine, was examined in more detail, conducting an inhibitor titration to obtain an IC_50_ of 9.32 ± 0.46 μM (Fig. 3e), comparable with previously reported values obtained under similar conditions (2.9 ± 0.5 μM).^45^ This was further corroborated using a commercial assay, ADP-Glo, generating an IC_50_ for staurosporine (Fig. S25†) of 15.3 ± 3.9 μM, consistent with the value obtained using **[Eu.1]^+^**. ADP-Glo functions by indirectly measuring the concentration of ADP formed in the kinase reaction. This involves running the reaction for a suitable time period, stopping the reaction and removing the unconsumed ATP, then converting the ADP to ATP and measuring its concentration by a chemiluminescent luciferase reaction. ADP-Glo serves as a good comparison to our assay because it measures the ADP formed; however, the measurements are not taken in real-time, and several steps need to be performed at certain time points to obtain reliable data. Consequently, ADP-Glo converts a 15 minute enzyme reaction into a 2 hour procedure. In comparison, our single Eu(iii) probe can monitor the reaction directly and continuously, enabling reliable kinetic parameters to be obtained more rapidly and with fewer steps, taking only the time of the enzyme reaction. These features offer the potential for increasing productivity and in drug discovery programmes, compared to current commercial assays.

### Monitoring the conversion between other NPP anions

The ability of **[Eu.1]^+^** to sense and differentiate other nucleoside polyphosphate anions (Fig. S26–S27†), offers the potential to monitor a wide variety of pharmaceutically important enzyme reactions, particularly those involving the formation of NDP or NMP anions, as these anions induce large enhancements in Eu(iii) emission intensity. To this end we simulated a range of NPP anion utilising enzymes, including NTPases, pyrophosphohydrolases and glycosyltransferases (Table 2, Fig. S28–S42†). In most cases these simulations gave a linear increase in the time-resolved Δ*J* = 2 emission intensity, implying the ability to profile a wide variety of NPP anion-utilising enzymes using a single luminescent probe.

**Table 2:** Range of enzyme reactions that can be monitored using **[Eu.1]^+^**, as determined by enzyme simulation experiments^*a*^

Enzyme class	Reaction	Nucleotide base	Other conditions tested	Figures^*b*^
Kinase	NTP → NDP	A, G, C, U, dA	See Table 1	S28
NTPase	NTP → NDP + Pi	A, G, C, U, dA	—	S29
NTP pyrophosphohydrolase	NTP → NMP + PPi	A, G, C, U	—	S30
NTP phosphohydrolase	NTP → NMP + 2 Pi	A, G, C, U	—	S31
Adenylate/guanylate/uridine/cytidine kinase	ATP + NMP → NDP + ADP	A, G, C, U	—	S32
Glycosyl transferase	UDP-sugar → UDP	Sugar = glucose, galactose	0.3–1.0 mM anion	4 and S34–S37,
			0–5 mM MgCl_2_	
			0–100 μM MnCl_2_	
Cyclic nucleotide phosphodiesterase	cNMP → NMP	A, G	0.3–1.0 mM anion	5 and S38–S42
			0–5 mM MgCl_2_	

^*a*^General conditions: 1 mM anion (substrate + product), 5 mM MgCl_2_ (2 mM for glycosyl transferase), 8 μM **[Eu.1]^+^**, 10 mM HEPES, pH 7.0, *λ*_exc_ = 292–366 nm, *λ*_em_ = 615–625 nm, integration time = 60–400 μs.

^*b*^See ESI.

To demonstrate the ability to monitor NPP anions possessing other nucleotide bases we used pyruvate kinase, which catalyses the conversion of ADP + phosphoenolpyruvate (PEP) to ATP + pyruvate but can use other NDP substrates. We monitored pyruvate kinase reactions (Fig. S33†) using different NDP anions (A, C, G, U and dA), observing the expected decrease in luminescence intensity (for all but CDP) over time as the NDP anion is converted to the corresponding NTP anion, dephosphorylating PEP in the process. Increasing the enzyme concentration gave a faster decrease in emission intensity, consistent with an increase in reaction rate. In the case of CDP, the decrease in **[Eu.1]^+^** emission intensity is comparable to that of the photobleaching experiment (in the absence of enzyme), consistent with CDP being a poor substrate for pyruvate kinase.^46^

### Monitoring glycosyltransferases

**Fig. 4:**
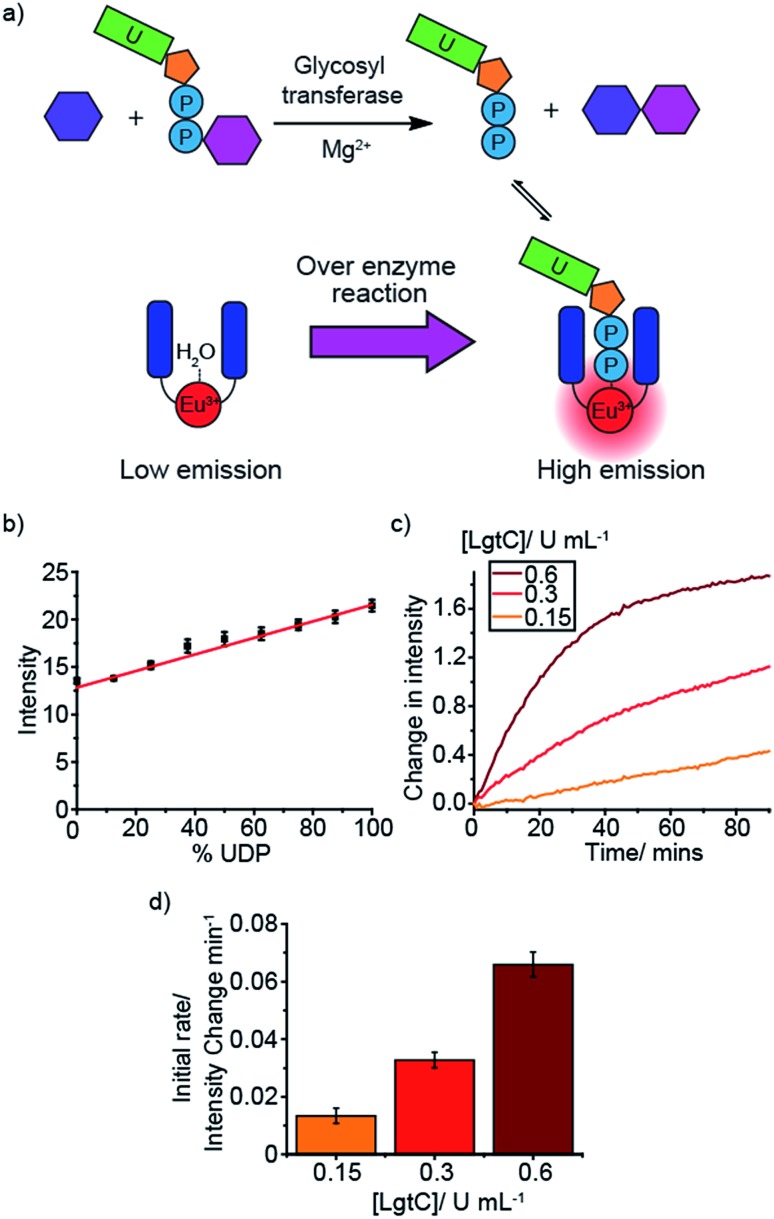
Real-time monitoring of a glycosyl transferase reaction. (a) Cartoon depicting the monitoring of glycosyl transferase activity using **[Eu.1]^+^** (b) LgtC simulation in standard assay conditions (1 mM UDP-galactose + UDP, 10 mM lactose, 2 mM MgCl_2_, 8 μM **[Eu.1]^+^**, 0.02% Triton X-100, 10 mM HEPES, pH 7.0, *λ*_exc_ = 292–366 nm,, *λ*_em_ = 615–625 nm, integration time = 60–400 μs), measuring the time-resolved luminescence intensity of differing ratios of UDP/(UDP-galactose + UDP) (% UDP). (c) and (d) Real-time monitoring of the LgtC catalysed transfer of galactose from UDP-galactose to lactose, generating UDP at different concentrations of LgtC using the time-resolved luminescence intensity of **[Eu.1]^+^** (c) and the calculation of the initial enzyme reaction rate (d). Conditions: 1 mM UDP-galactose, 2 mM MgCl_2_, 10 mM lactose, 8 μM **[Eu.1]^+^**, 0.02% Triton X-100, 10 mM HEPES, pH 7.0, *λ*_exc_ = 292–366 nm, *λ*_em_ = 615–625 nm, integration time = 60–400 μs.

An important enzyme class for biological and therapeutic applications is the glycosyltransferases (GTs). GTs catalyse the transfer of saccharide units from a glycosyl donor (*e.g.* a UDP-sugar) to an acceptor (*e.g.* oligosaccharide) (Fig. 4a).^3^,^4^ GTs are involved in a range of biological processes underpinning health and disease, including cell signalling, adhesion and cell wall biosynthesis, and thus represent attractive druggable targets. However, progress in GT inhibitor development is restricted by the lack of label-free tools for conducting HTS assays. We simulated GT reactions using two different glycosyl donors, UDP-glucose and UDP-galactose, which are converted to UDP during the reaction (Fig. 4b and S34–S37†). We used glucose and maltose as example acceptor and product sugars, having shown that various mono- and disaccharides at 10 mM concentration have very little effect on the luminescence of **[Eu.1]^+^** (Fig. S43†). Varying the ratio of UDP/UDP-sugar in the presence of **[Eu.1]^+^** (Fig. 4b) gave a linear increase in emission as the mole fraction of UDP increases. The simulated reaction was conducted in a range of different conditions (Fig. S34–S37†), varying the concentration of anion (0.3–1.0 mM), Mg^2+^ ions (0–5 mM) and Mn^2+^ ions (0–100 μM), common GT cofactors, demonstrating the potential to correlate the emission of **[Eu.1]^+^** to the progress of a GT reaction.

Next, the probe was used to monitor the activity of the bacterial glycosyltransferase, LgtC, a promising target for the discovery of novel antibiotics. LgtC catalyses the transfer of galactose from UDP-galactose to a lactose acceptor, producing UDP in the process. When this reaction is performed in the presence of **[Eu.1]^+^** there is an increase in the time-resolved luminescence intensity of the Δ*J* = 2 band over time (Fig. 4c). Moreover, the initial rates of the reaction depended linearly on the concentration of enzyme used (Fig. 4d). This demonstrates the ability of **[Eu.1]^+^** to monitor another important class of NPP-utilising enzymes.

Next, the probe was used to monitor the activity of the bacterial glycosyltransferase, LgtC, a potential target for the discovery of novel antibiotics. LgtC catalyses the transfer of galactose from UDP-galactose to a lactose acceptor, producing UDP in the process. When this reaction is performed in the presence of **[Eu.1]^+^** there is an increase in the time-resolved luminescence intensity of the Δ*J* = 2 band over time (Fig. 4c). Moreover, the initial rates of the reaction depended linearly on the concentration of enzyme used (Fig. 4d). This demonstrates the ability of **[Eu.1]^+^** to monitor another important class of NPP-utilising enzymes.

### Monitoring phosphodiesterases

**Fig. 5:**
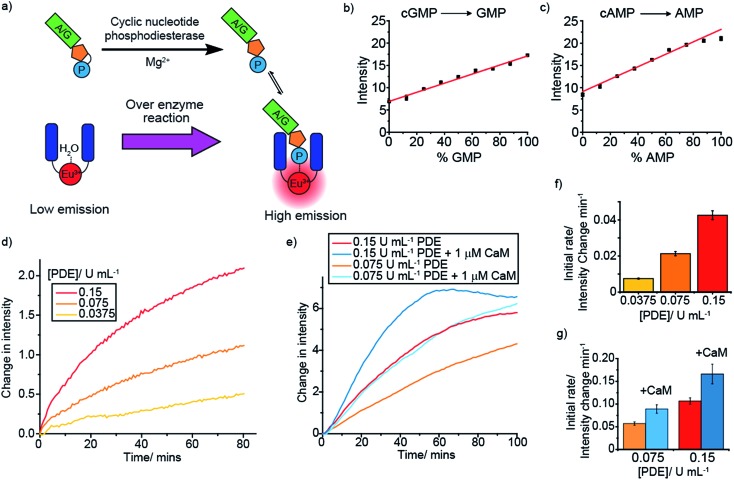
Real-time monitoring of phosphodiesterase activity. (a) Cartoon depicting the monitoring of cyclic nucleotide phosphodiesterase using **[Eu.1]^+^**, (b) and (c) Simulations of the phosphodiesterase reactions, cGMP to GMP (b) and cAMP to AMP (c), measuring the time-resolved luminescence intensity of **[Eu.1]^+^** with increasing NMP/(cNMP + NMP) ratio (% NMP). Conditions: 1 mM cNMP + NMP, 5 mM MgCl_2_, 8 μM **[Eu.1]^+^**, 10 mM HEPES, pH 7.0, *λ*_exc_ = 292–366 nm, *λ*_em_ = 615–625 nm, integration time = 60–400 μs. (d)–(g) Real-time monitoring of the PDE-catalysed conversion of cGMP to GMP (d) and cAMP to AMP with calmodulin (CaM) activation (e), using the time-resolved luminescence intensity of **[Eu.1]^+^**, and comparing the change in initial rate on changing concentration of enzyme (f) and addition of calmodulin (g). Conditions: 1 mM cNMP, 5 mM MgCl_2_, 0.03 mM CaCl_2_ (d and e), 8 μM **[Eu.1]^+^**, 10 mM HEPES, pH 7.0, *λ*_exc_ = 292–366 nm, *λ*_em_ = 615–625 nm, integration time = 60–400 μs.

Cyclic nucleotide phosphodiesterases (PDEs) represent another class of drug target that are difficult to screen. Cyclic nucleotide PDEs catalyse the conversion of the key signalling molecules, cAMP and cGMP, to AMP and GMP (Fig. 5a), and hence are important in cell signalling.^47^ Having shown previously that **[Eu.1]^+^** binds relatively strongly to AMP (log *K*_a_ = 3.4), and that both AMP and GMP induce distinctive emission responses compared to those of cAMP and cGMP, we decided to simulate each of these PDE reactions. Upon increasing the NMP/cNMP ratio there is a linear increase in time-resolved emission intensity of **[Eu.1]^+^** (Fig. 5b and c). Incubating each of the cNMP anions with a PDE enzyme in the presence of **[Eu.1]^+^** resulted in the expected increase in luminescence over time, with increasing rates observed with increasing enzyme concentration (Fig. 5d and f and S44†). Furthermore, upon addition of phosphodiesterase activators, Ca^2+^ ions and calmodulin,^48^ a faster rate of **[Eu.1]^+^** emission intensity increase and hence enzyme reaction rate was observed (Fig. 5e and g), as anticipated on addition of an enzyme activator.

### Monitoring sequential enzyme reactions

**Fig. 6:**
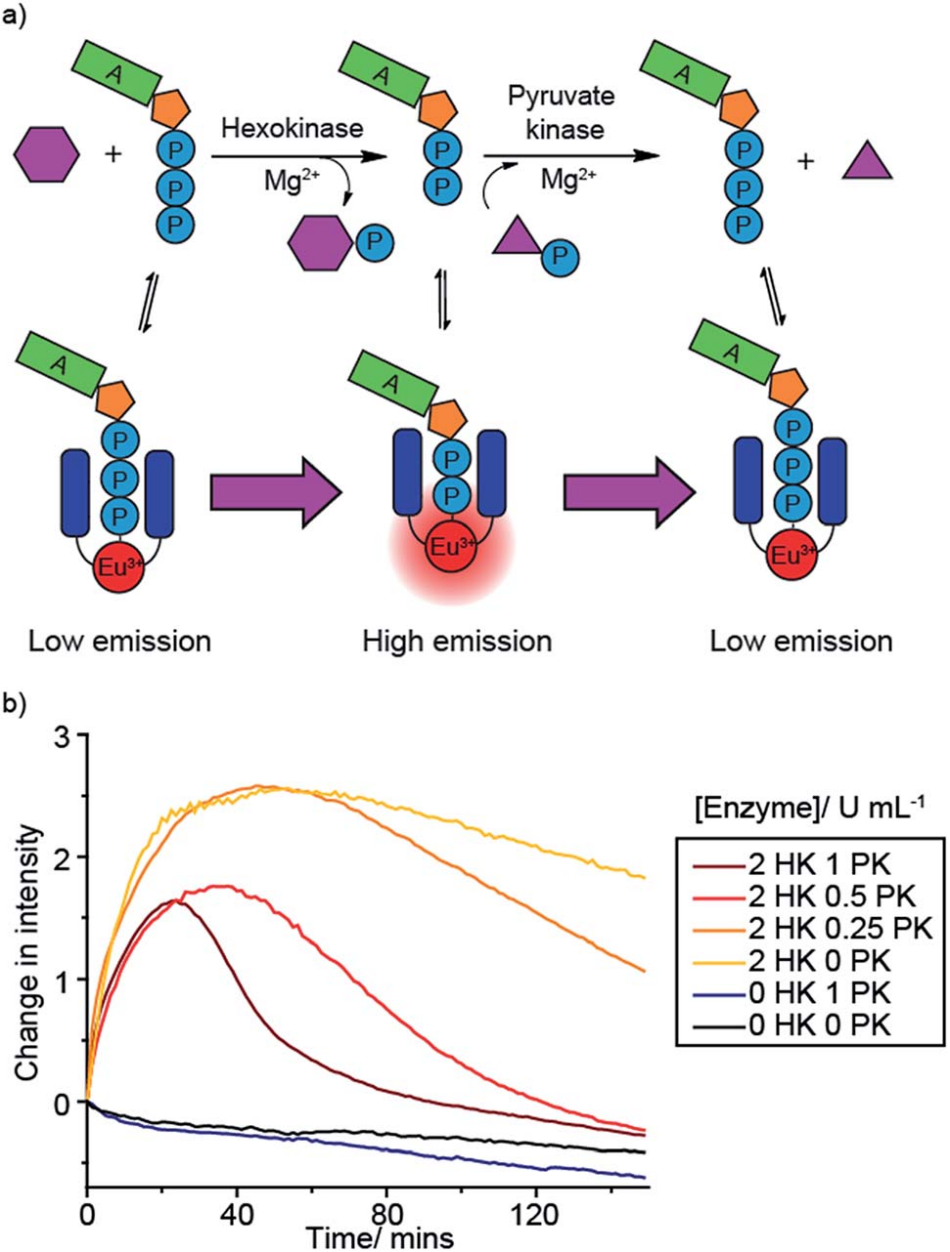
Real-time monitoring of sequential enzyme reactions, involving hexokinase (HK, ATP to ADP) and pyruvate kinase (PK, ADP to ATP). (a) Cartoon depicting sequence of enzyme monitoring using **[Eu.1]^+^**. (b) Real-time monitoring of hexokinase followed by pyruvate kinase using the time-resolved emission of **[Eu.1]^+^**. Conditions: 1 mM ATP, 1 mM glucose, 1 mM PEP, 5 mM MgCl_2_, 50 mM KCl, 8 μM **[Eu.1]^+^**, 10 mM HEPES, pH 7.0, *λ*_exc_ = 292–366 nm, *λ*_em_ = 615–625 nm, integration time = 60–400 μs).

A benefit of monitoring enzyme reactions in real-time, rather than using single-point detection, is that we can monitor sequential enzyme reactions, enabling a biosynthetic pathway to be followed. This is important not only in drug discovery but also in large-scale biosynthesis, where knowledge of the optimum time to add enzymes and substrates is crucial for maximising product yield and process efficiency. To demonstrate the principle of monitoring sequential enzyme reactions, we used two previously studied enzymes, hexokinase (HK) and pyruvate kinase (PK), which are involved in the same metabolic pathway, glycolysis. Hexokinase catalyses the conversion of ATP to ADP during the phosphorylation of glucose, while pyruvate kinase converts ADP back to ATP (along with dephosphorylation of PEP) (Fig. 6a). We monitored this reaction sequence in one pot using **[Eu.1]^+^**. Initially the emission intensity increased as ATP is converted to ADP by hexokinase, followed by a decrease in luminescence as ADP is converted back to ATP by pyruvate kinase (Fig. 6b).

Decreasing the concentration of pyruvate kinase decreased the rate of conversion of ADP back to ATP and hence the rate of decrease in Eu(iii) luminescence, as well as yielding a higher maximal intensity response. In the absence of hexokinase there is very little change in luminescence compared to the bleaching experiment (no hexokinase nor pyruvate kinase present), whereas in the absence of pyruvate kinase but presence of hexokinase (yellow line) there is a rapid initial intensity increase as the hexokinase converts ATP to ADP, followed by a plateauing of the signal as the ADP cannot be converted back to ATP (some photobleaching is observed in this case). Reversing the enzyme reaction sequence, by starting with ADP and monitoring its conversion to ATP by pyruvate kinase, followed by the hexokinase catalysed conversion of ADP back to ATP, gave the opposite luminescence response: an initial decrease followed by an increase in **[Eu.1]^+^** emission (Fig. S45†). These experiments highlight the capability of our supramolecular enzyme assay for monitoring sequential enzyme reactions in one solution, a feature that is not possible using commercial end-point assay technologies.

## Conclusions

In conclusion, we have demonstrated that the phosphoanion binding and sensing properties of a single cationic Eu(iii) complex, **[Eu.1]^+^**, can be exploited to develop a versatile assay platform for real-time monitoring of a range of pharmaceutically important enzymes, including kinases, glycosyltransferases and phosphodiesterases. The ability of **[Eu.1]^+^** to discriminate between nucleoside tri-, di- and monophosphate anions allowed the enzymatic production of different NPP anions to be monitored in a convenient luminescence-based increase-in-signal format.

The robustness of our assay platform is illustrated by its ability to operate in a wide-range of biologically relevant conditions, determine accurate kinetic parameters (*K*_m_, IC_50_) and monitor sequential enzyme reactions. Our Eu(iii) probe offers significant advantages over existing assay technologies, including: (1) label- and antibody-free detection; (2) a time-resolved luminescent signal that eliminates background fluorescence; and (3) real-time measurements that enable both qualitative and quantitative analysis of enzyme activity, selectivity and inhibition. Moreover, our microplate assay can be performed at physiological concentrations of NPP anions (*e.g.* 1–5 mM ATP for kinase assay) and thus can be used to screen for inhibitors of low activity enzymes, a feature that is not possible using current assay platforms.

By utilising a single molecular probe, our assay has the potential to simplify HTS screening processes significantly, extending the breadth of substrate-specific assays, and allowing a wide range of enzymes and substrates to be screened under identical assay conditions. Moreover, the ability to study natural substrates could facilitate a better understanding of enzyme inhibition mechanism, reducing the possibility of drug candidates passing early development stages, only to fail at a later, more costly phase.

## Conflicts of interest

There are no conflicts to declare.

## Supplementary Material

Supplementary information
