# Supplementary material for: A simple, robust, universal assay for real-time enzyme monitoring by signalling changes in nucleoside phosphate anion concentration using a europium(iii)-based anion receptor
Source: Chem Sci. 2019 May 1;10(20):5373–81. doi: 10.1039/c9sc01552c (PMC6540902; doi:10.1039/c9sc01552c)
Supplement: Supplementary file 1 [file SC-010-C9SC01552C-s001.pdf]

Electronic Supplementary Material (ESI) for Chemical Science.
This journal is © The Royal Society of Chemistry 2019

Supporting Information

A simple, robust, universal assay for real-time enzyme monitoring by signaling changes in nucleoside phosphate anion concentration using a europium(III)-based anion receptor

Sarah H. Hewitt[†], Rozee Ali[†], Romain Mailhot[†], Chloe Antonen[†], Charlotte A. Dodson[‡] and Stephen J. Butler[†]*

[†]*Department of Chemistry, Loughborough University, Epinal Way, Loughborough, LE11 3TU, UK.*

[‡]*Department of Pharmacy & Pharmacology, University of Bath, Claverton Down, Bath, BA2 7AY, UK.*

General Considerations

All chemicals used were purchased from standard chemical suppliers and used without further purification. [**Eu.1**]+ was synthesised using a synthetic protocol described previously.[1]

Measurements of pH were carried out using a Jenway 3510 pH/mV meter with a Jenway combination electrode or a Jenway 3020 pH meter with an Aldrich glass combination pH electrode, both calibrated using buffer solutions of pH 4.00 ± 0.01, 7.00 ± 0.01 and 10.00 ± 0.01.

1.1 Optical Spectroscopy

UV/Vis absorbance spectra were measured using a Shimadzu UV-1800 UV-spectrophotometer.

Emission spectra were recorded on a SPEX Fluoromax luminescence spectrometer using dM300 version 3.12 software. Emission spectra were obtained using a 40 μL Hellma Analytics quartz cuvette (Art no. 111-10-K-40). Excitation light was set at 330 nm, and emission read in the range 550 – 720 nm (for titrations) or 570 – 635 nm (for enzyme assays), using an integration time of 0.5 seconds, increment of 0.5 nm and excitation and emission slits of 0.5 nm.

Plate reader data was obtained on a BMG Labtech CLARIOstar microplate reader in black Fisherbrand™ 384-well plates, using a total volume of 40 μL per well.

1.2 Biophysical measurement procedures

All biophysical analyses were carried out in degassed 10 mM HEPES, pH 7.0 buffer, unless otherwise stated. Concentrated [**Eu.1**]+ stocks were made at ~2.5 mg mL^{-1} in methanol prior to diluting to 80 μM in 10 mM HEPES, pH 7.0 and the accurate concentration determined using the UV/Vis absorbance at 332 nm (ε = 12 500 M^{-1} cm^{-1})[1] in 10 mM HEPES, pH 7.0. Unless otherwise stated, the [**Eu.1**]+ concentration was kept at 8 μM concentration. Stocks of phosphoanions were made up at 5 or 25 mM and adjusted to pH 7.0 by addition of minimal volumes of 1 M NaOH or 1 M HCl. Stocks of $MgCl_2$ and DTT were made up at 50 or 250 mM in 10 mM HEPEs, pH 7.0. Stocks of sugars (glucose, maltose, mannose, fructose and lactose) were made up at 100 mM.

Anion screen

Emission spectra of 50 μL of [**Eu.1**]+ (8 μM) with the phosphoanions (1 mM), or buffer were taken. 1 μL of 250 mM $MgCl_2$ was added, and the emission spectrum taken again.

Fluorometer-based enzyme simulation

Variable amounts of an appropriate solution of enzyme product, containing the phosphoanion (generally 1 mM), $MgCl_2$ (generally 5 mM), 8 μM [**Eu.1**]$^+$, and any other stated components in 10 mM HEPES (pH 7.0) was added to 50 μL of a solution of enzymatic substrate, containing the phosphoanion (generally 1 mM), $MgCl_2$ (generally 5 mM), [**Eu.1**]$^+$ (8 μM) and any other stated components in 10 mM HEPES (pH 7.0). Emission spectra (λ_{exc} = 330 nm, λ_{em} = 550 – 720 nm) were recorded after each addition. The ratios of the intensity at 616.5 nm to 599.5 nm (or stated) were taken and plotted against the percentage of enzyme product in the cuvette.

Plate-based enzyme simulation

Differing ratios of a solution of enzyme product or substrate, containing the phosphoanion (generally 1 mM), $MgCl_2$ (generally 5 mM), [**Eu.1**]$^+$ (generally 8 μM) and other state components in 10 mM HEPES (pH 7.0) to a total volume of 40 μL were added to a 384-well plate, in triplicate, and the plate incubated for 30 minutes prior to reading. Luminescence intensities were taken with excitation at 292 – 366 nm and emission using time-resolved measurements at 615 – 625 nm using an integration time of 60 – 400 μs or intensity at 601-633 nm. The mean of the triplicate intensity values were taken and plotted against the percentage enzyme product in the well. Error bars indicate the standard error in mean.

General hexokinase enzyme assay

To 20 μL of a solution containing ATP (2 mM or 2× final concentration), $MgCl_2$ (10 mM or 2× final concentration), **[Eu.1]**$^+$ (16 μM or 2× final concentration) and stated other components (at 2× final concentration) in 10 mM HEPES, pH 7.0 in a 384-well plate was added 10 μL of a solution of hexokinase (at 4× final concentration) or buffer, and the plate incubated for 15 minutes. 10 μL of a glucose (or appropriate other sugar, at 4× final concentration) was added and the plate read immediately. Luminescence intensities were taken with excitation at 292 – 366 nm and emission using time-resolved measurements at 615 – 625 nm using an integration time of 60 – 400 μs or intensity at 601-633 nm, at appropriate time intervals (often 30 s). Assays were run in triplicate and the mean of the triplicate intensity values were taken and plotted against time. Where appropriate bleaching (no enzyme) experiments were taken away from experiments containing hexokinase.

Hexokinase different concentrations of ATP, $MgCl_2$ or fructose

A titration using a 2/3 dilution regime was used to vary the concentrations, the protocol is detailed below.

20 μL of buffer was added to 16 wells in one row of a 384-well plate. 40 μL of an appropriate ATP, $MgCl_2$ or fructose solution was added to the first well and mixed. 40 μL was transferred from the first well to the second well and mixed. This process was repeated to the 15th well, where the 40 μL was discarded, and the 16th well left with just buffer. 10 μL of a solution containing hexokinase (at 4× final concentration), 32 μM **[Eu.1]**$^+$, $MgCl_2$ (20 mM if appropriate) and ATP (4 mM if appropriate) was added to each well and the plate incubated for 15 minutes. 10 μL of a solution of ATP (4 mM) or glucose (40 mM) as appropriate was added and the plate read immediately. Luminescence intensities were taken with excitation at 292 – 366 nm and emission using time-resolved measurements at 615 – 625 nm using an integration time of 60 – 400 μs or at appropriate time intervals (often 30 s). Assays were run in triplicate and the mean of the triplicate intensity values were taken and plotted against time.

For the fructose Michaelis-Menten calculations the initial rate was calculated as the gradient of the intensity against time over the first 5 minutes for each measurement. These initial rates were plotted against fructose concentration and fit to a Michaelis-Menten equation

using Origin 2015.

Aurora A enzyme assay

Aurora A enzyme assays were performed in a buffer containing 50 mM NaCl, 5 mM $MgCl_2$, 0.25 mM DTT, 2.5 % glycerol in 10 mM HEPES, pH 7.0. To a 20 μL solution containing kemptide (2 mM) and **[Eu.1]**$^+$ (16 μM) was added 10 μL of an appropriate Aurora A solution in a 384-well plate. The wells were mixed and the plate incubated for 15 minutes. 10 μL of ATP (4 mM) was added and the plate read immediately. Luminescence intensities were taken with excitation at 292 – 366 nm and emission using time-resolved measurements at 615 – 625 nm using an integration time of 60 – 400 μs, at appropriate time intervals. Assays were run in triplicate and the mean of the triplicate intensity values were taken and plotted against time. Where appropriate bleaching (no enzyme) experiments were taken away from experiments containing Aurora A.

Kemptide Michaelis-Menten

A titration using a 2/3 dilution regime was used to vary the kemptide concentration, the protocol is detailed below. These assays were performed in buffer containing 50 mM NaCl, 5 mM $MgCl_2$, 0.25 mM DTT, 2.5 % glycerol in 10 mM HEPES, pH 7.0

20 µL of buffer was added to 16 wells in one row of a 384-well plate. 40 µL of kemptide (4.5 mM) was added to the first well and mixed. 40 µL was transferred from the first well to the second well and mixed. This process was repeated to the 15th well, where the 40 µL was discarded, and the 16th well left with just buffer. 10 µL of a solution containing Aurora A (4 µM) and **[Eu.1]**$^{+}$ (32 µM), was added to each well and the plate incubated for 15 minutes. 10 µL of a solution of ATP (4 mM) was added and the plate read immediately. Luminescence intensities were taken with excitation at 292 – 366 nm and emission using time-resolved measurements at 615 – 625 nm using an integration time of 60 – 400 µs or intensity at 601- 633 nm, at appropriate time intervals (often 75 s). Assays were run in triplicate and the mean of the triplicate intensity values were taken and plotted against time. For Michaelis-Menten calculations the initial rate was calculated as the gradient of the intensity against time over the first 25% of the enzyme reaction. These initial rates were plotted against kemptide concentration and fit to a Michaelis-Menten equation using Origin 2015.

Aurora A single point inhibition/activation assay

To 20 µL of kemptide (1 mM), Aurora A (2 µM or 0 µM), and **[Eu.1]**$^{+}$ (16 µM) in 50 mM NaCl, 5 mM MgCl2, 0.25 mM DTT, 2.5 % glycerol in 10 mM HEPES, pH 7.0 was added 10 µL of the inhibitor/activator (4 µM, 2 µM or 0 µM) in 10% DMSO (0% for TPX2 activation) in a 384-well plate. The wells were mixed and incubated for 15 minutes prior to reading. Immediately before reading 10 µL of ATP (4 mM) was added to each well. Luminescence intensities were taken with excitation at 292 – 366 nm and emission using time-resolved measurements at 615 – 625 nm using an integration time of 60 – 400 µs. Assays were run in triplicate and the mean of the triplicate intensity values were taken and plotted against time, and the control with no enzyme intensity taken away. Initial rates were calculated by the gradient of the intensity against time over the first 5 minutes (3 minutes for activation or no inhibitor) of the enzyme reaction.

Staurosporine IC_{50}

A titration using a 2/3 dilution regime was used to vary the staurosporine concentration, the protocol is detailed below.

20 μL of 10% DMSO in buffer was added to wells 2 to 16 of one row of a 384-well plate. 60 μL of staurosporine (200 μM), 10% DMSO in buffer was added to the 1st well. 40 μL was taken from the 1st well, and transferred to the 2nd well and mixed. This was repeated down to the 15th, where the 40 μL was discarded and the 16th well left without any staurosporine. 10 μL of kemptide (2 mM), Aurora A (200 nM) and **[Eu.1]⁺** (32 μM) was added to each well, the wells mixed and the plate incubated for 15 minutes. Immediately before reading 10 μL of ATP (4 mM) was added to each well. Luminescence intensities were taken with excitation at 292 – 366 nm and emission using time-resolved measurements at 615 – 625 nm using an integration time of 60 – 400 μs. Assays were run in triplicate and the mean of the triplicate intensity values were taken and plotted against time. Initial rates were calculated by the change in intensity against time over the first 15 minutes, plotted against staurosporine concentration and fitted to a sigmoidal curve to calculate the IC_{50}.

ADP-Glo assay (staurosporine IC_{50})

A 1 in 2 dilution regime was used to vary the concentration of staurosporine. 20 μL of 10% DMSO in buffer was added to wells 2 to 8 of one row of a 384-well plate. 40 μL of staurosporine (200 μM) in 10% DMSO in buffer was added to the 1st well. 20 μL was taken from the 1st well, and transferred to the 2nd well and mixed. This was repeated down to the 8th, where the 20 μL was discarded. 10 μL of kemptide (2 mM) and Aurora A (200 nM) was added to each well, the wells mixed and the plate incubated for 15 minutes. 10 μL of ATP (4 mM) was added to each well, and the plate incubated for 15 minutes. 15 μL of the enzyme reaction solution was transferred to a 96-well Sterilin white plate containing 15 μL of ADP-Glo reagent in appropriate wells. The wells were mixed and the plate incubated for 40 minutes. 30 μL of ADP-Glo detection reagent was added to the wells, the wells were mixed and the plate incubated for 1 hour. The plate was read by reading the full luminescence emission. Assays were run in triplicate, with a duplicate calibration line of varying ATP:ADP ratios, which was used to calculate the concentration of ADP in the wells. Initial rates were calculated as the [ADP] formed over the 15 minute enzyme reaction, and the mean of the triplicate values calculated. The initial rates were plotted against staurosporine concentration and fitted to a sigmoidal curve to calculate the IC_{50}.

LgtC enzyme assay

To 20 μL of a solution containing UDP-galactose (2 mM), $MgCl_2$ (4 mM), **[Eu.1]⁺** (16 μM) and 0.02% Triton-X 100 in 10 mM HEPES, pH 7.0 in a 384-well plate was added 10 μL of a solution

of LgtC (2.4, 1.2, 0.6 or 0 U mL^{-1}), and the plate incubated for 15 minutes. 10 µL of lactose (40 mM) was added and the plate read immediately. Luminescence intensities were taken with excitation at 292 – 366 nm and emission using time-resolved measurements at 615 – 625 nm using an integration time of 60 – 400 µs, at appropriate time intervals (45 s). Assays were run in triplicate and the mean of the triplicate intensity values were taken and plotted against time. Bleaching (no enzyme) experiments were taken away from experiments containing LgtC.

PDE enzyme assay

To 20 µL of a solution containing $MgCl_2$ (10 mM), **[Eu.1]**$^+$ (16 µM) and if appropriate, calmodulin (2 µM) and $CaCl_2$ (60 µM) in 10 mM HEPES, pH 7.0 in a 384-well plate was added 10 µL of a solution of phosphodiesterase (0.6, 0.3, 0.15 or 0 U mL^{-1}), and the plate incubated for 15 minutes. 10 µL of cAMP or cGMP (4 mM) was added and the plate read immediately. Luminescence intensities were taken with excitation at 292 – 366 nm and emission using time-resolved measurements at 615 – 625 nm using an integration time of 60 – 400 µs, at appropriate time intervals. Assays were run in triplicate and the mean of the triplicate intensity values were taken and plotted against time. Bleaching (no enzyme) experiments were taken away from experiments containing PDE. Initial rates for the calmodulin experiments were calculated as the gradient of intensity over time for the first 30% of the enzyme reaction.

Simultaneous enzyme reactions

20 µL of a solution containing $MgCl_2$ (10 mM), **[Eu.1]**$^+$ (16 µM) and ATP or ADP (2 mM), as appropriate, in 10 mM HEPES, pH 7.0, was added to a 384-well plate. 5 µL of an appropriate hexokinase solution and 5 µL of an appropriate pyruvate kinase solution were added. The wells were mixed and incubated for 10 minutes. 10 µL of a solution containing phosphor(enol)pyruvate (4 mM) and glucose (4 mM) was added to all the wells and the plate read immediately. Luminescence intensities were taken with excitation at 292 – 366 nm and emission using time-resolved measurements at 615 – 625 nm using an integration time of 60 – 400 µs, at appropriate time intervals. Assays were run in triplicate and the mean of the triplicate intensity values were taken and plotted against time.

Anion screen

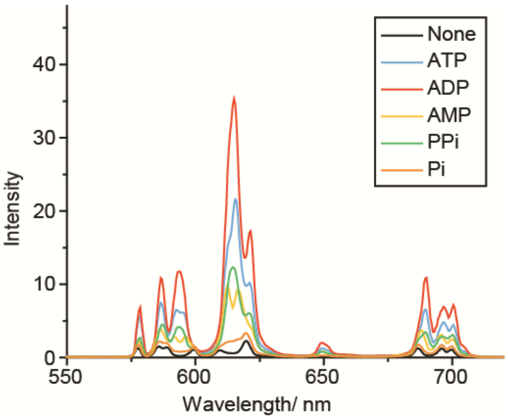

Figure S1. Effect of the addition of phosphoanions (1 mM) on the emission spectra of [**Eu.1**]$^+$. Conditions: 8 μM [**Eu.1**]$^+$, 1 mM phosphoanion, 10 mM HEPES, pH 7.0, λ_{exc} = 330 nm

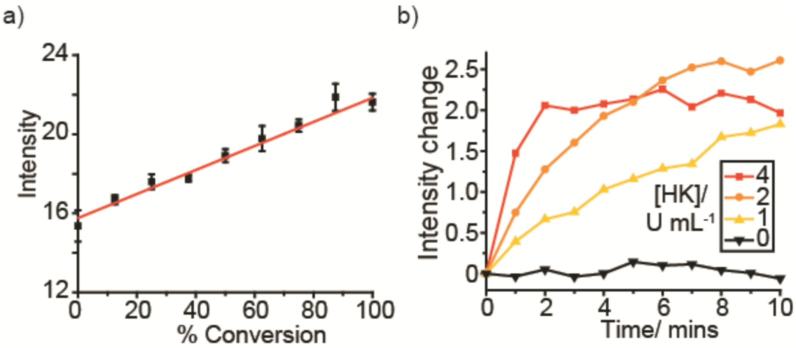

Figure S2. Plate-based real-time monitoring of a kinase reaction. a) Kinase simulation in standard assay conditions (1 mM ATP+ADP, 5 mM $MgCl_2$, 8 μM **[Eu.1]$^+$**, 10 mM HEPES, pH 7.0), measuring the emission intensity (λ_{exc} = 615 – 625 nm, λ_{em} = 601 - 631 nm) of differing ratios of ATP/ADP (% conversion of ATP to ADP). b) Real-time monitoring of a model kinase (hexokinase) using the time-resolved luminescence intensity of **[Eu.1]$^+$**, conditions: 1 mM ATP, 5 mM $MgCl_2$, 10 mM glucose, 8 μM **[Eu.1]$^+$**, 10 mM HEPES, pH 7.0, λ_{exc} = 292 – 366 nm, λ_{em} = 601 - 631 nm.

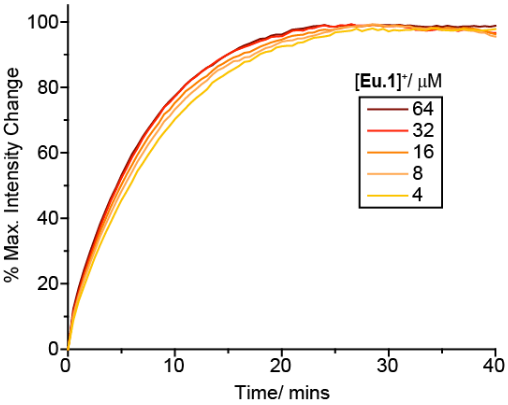

Figure S3. Real-time monitoring of the hexokinase catalysed phosphorylation of glucose at different concentrations of [**Eu.1**]$^+$. Conditions: 1 mM ATP, 10 mM glucose, 1 – 16 μM [**Eu.1**]$^+$, 5 mM $MgCl_2$, 10 mM HEPES, pH 7.0, λ_{exc} = 292 – 366 nm, λ_{em} = 615 - 625 nm, integration time 60 – 400 μs, measurements taken every 30 s, average of triplicate reactions

Different [ATP/ADP]

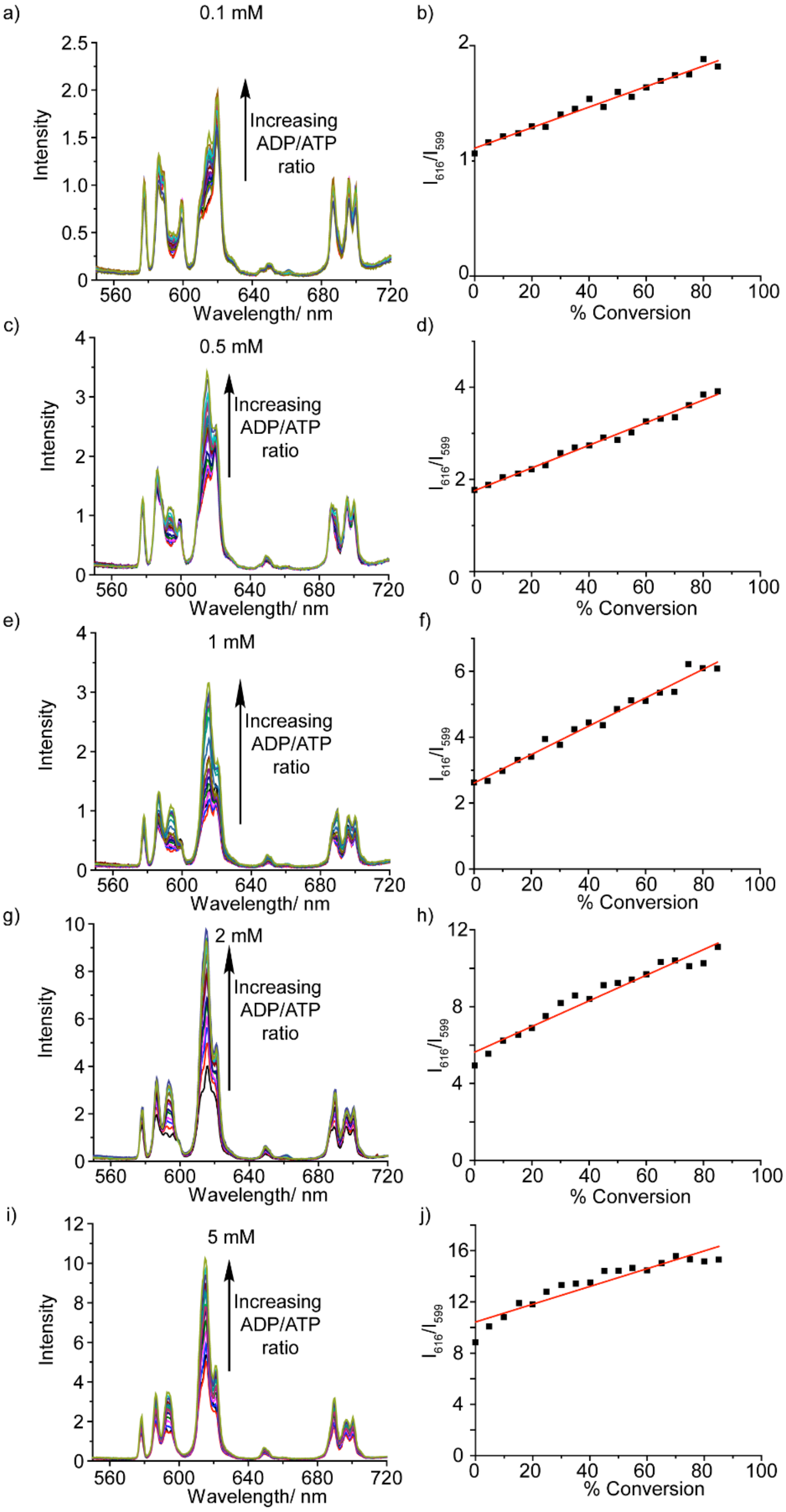

Figure S4. Fluorometer-based kinase simulation at different [anion]: 0.1 mM (a) and b)), 0.5 mM (c) and d)), 1 mM (e) and f)), 2 mM (g) and h)) and 5 mM (i) and j) ATP or ADP. a), c), e), g) and i) Emission spectra of [**Eu.1**]$^{+}$ on increasing ADP/ATP ratio, b), d), f), h) and j) Plot of emission intensity ratio 616/599 nm versus percentage conversion of ATP to ADP. 8 μM [**Eu.1**]$^{+}$, variable [ATP+ADP], 5 mM $MgCl_2$, 10 mM HEPES, pH 7.0, λ_{exc} = 330 nm

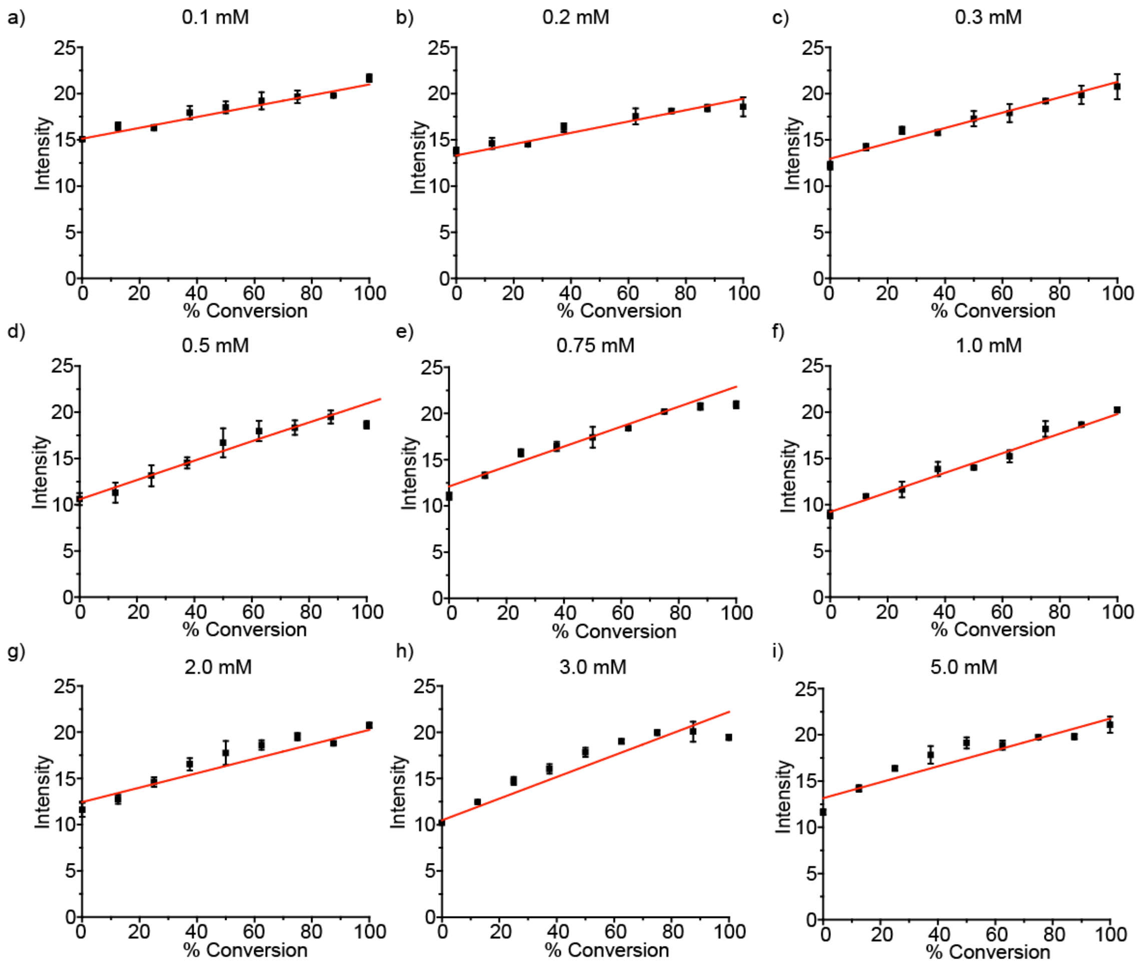

Figure S5. Plate-based kinase simulation using time-resolved method with different anion (ATP+ADP) concentrations: 0.1 (a), 0.2 (b), 0.3 (c), 0.5(d), 0.75 (e), 1.0 (f), 1.5 (g), 2.0 (h), 3.0 (i) and 5.0 (j) mM [ATP] + [ADP]. 8 μM [**Eu.1**]$^+$, variable [ATP+ADP], 5 mM $MgCl_2$, 10 mM HEPES, pH 7.0, λ_{exc} = 292 – 366 nm, λ_{em} = 615 - 625 nm, integration time 60 – 400 μs

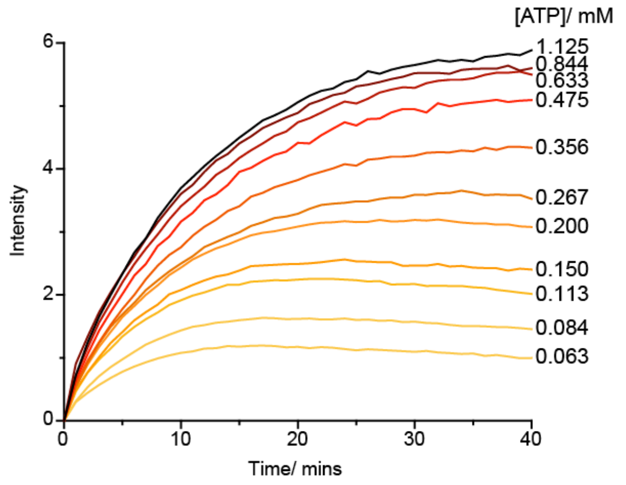

Figure S6. Real-time monitoring of the hexokinase catalysed phosphorylation of glucose at different concentrations of ATP. Conditions: variable ATP, 10 mM glucose, 8 μM [**Eu.1**]$^+$, 5 mM $MgCl_2$, 0.2 U mL^{-1} hexokinase, 10 mM HEPES, pH 7.0, λ_{exc} = 292 – 366 nm, λ_{em} = 615 - 625 nm, integration time 60 – 400 μs, measurements taken every 60 s, average of triplicate reactions

Different [$MgCl_2$]

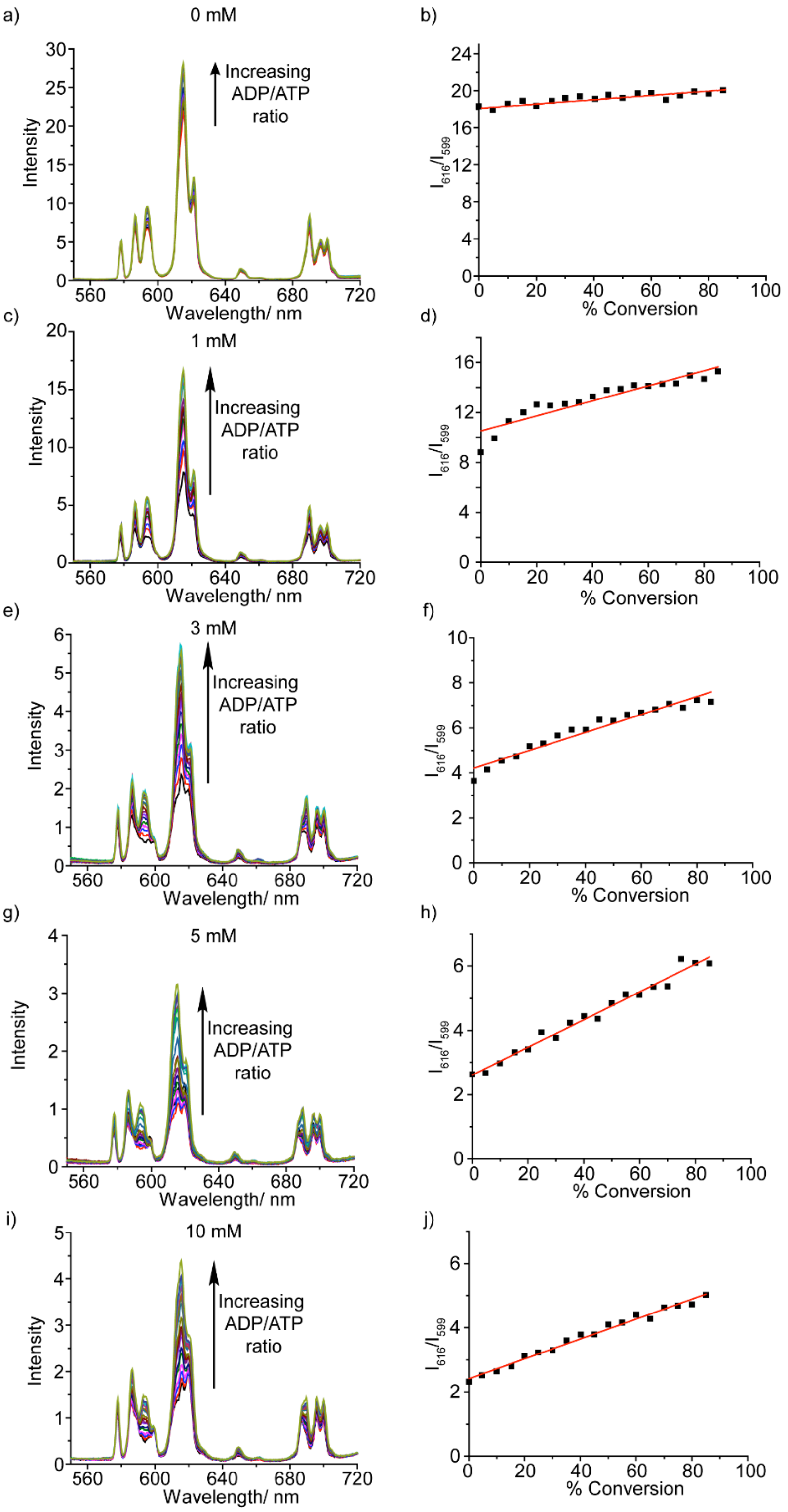

Figure S7. Fluorometer-based kinase simulation at different [$MgCl_2$]: 0 mM (a) and b)), 1 mM (c) and d)), 3 mM (e) and f)), 5 mM (g) and h)) and 10 mM (i) and j) $MgCl_2$. a), c), e), g) and i) Emission spectra of [**Eu.1**]$^+$ on increasing ADP/ATP ratio, b), d), f), h) and j) Plot of emission intensity ratio 616/599 nm versus percentage conversion of ATP to ADP. 8 μM [**Eu.1**]$^+$, 1 mM ATP+ADP, 0 – 10 mM $MgCl_2$, 10 mM HEPES, pH 7.0, λ_{exc} = 330 nm

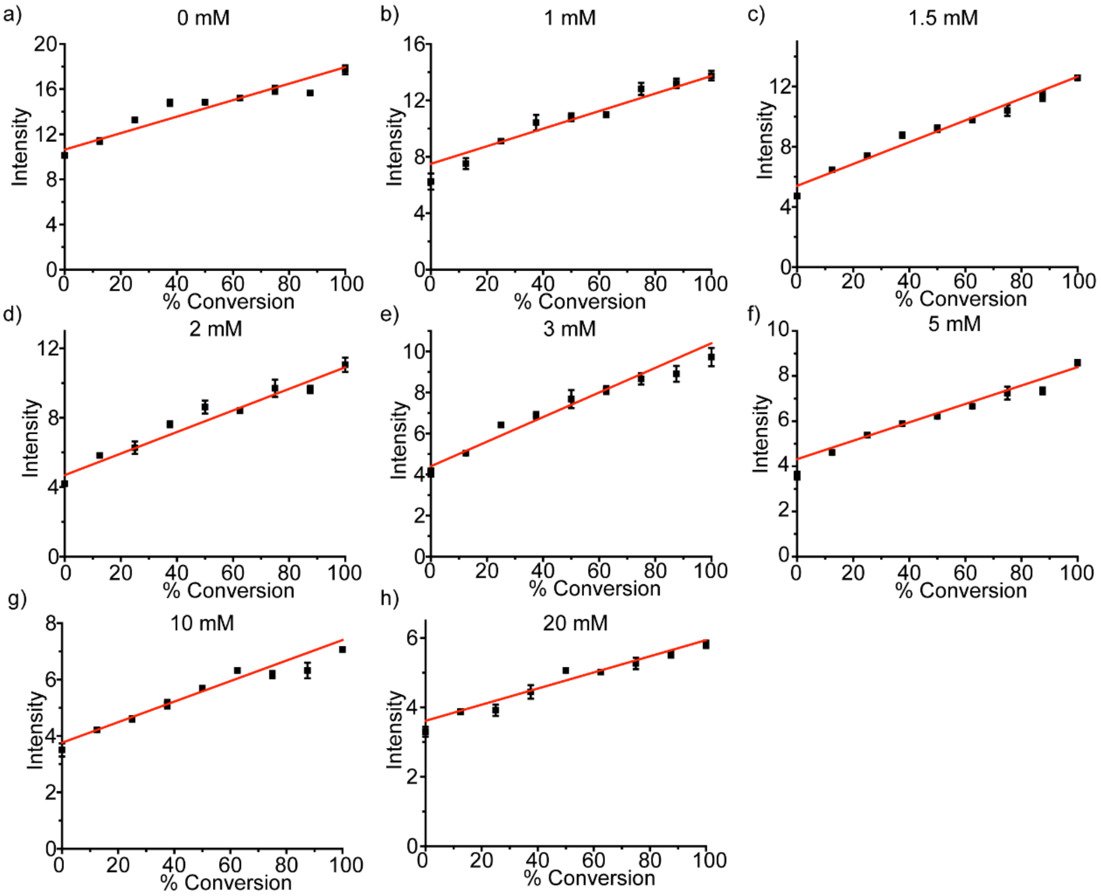

Figure S8. Plate-based kinase simulation using time-resolved method with different $MgCl_2$ concentrations: 0 (a), 1 (b), 1.5 (c), 2 (d), 3 (e), 5 (f), 10 (g), and 20 (h) mM $MgCl_2$. 8 μM [**Eu.1**]$^+$, 1 mM [ATP+ADP], variable $MgCl_2$, 10 mM HEPES, pH 7.0, λ_{exc} = 292 – 366 nm, λ_{em} = 615 - 625 nm, integration time 60 – 400 μs

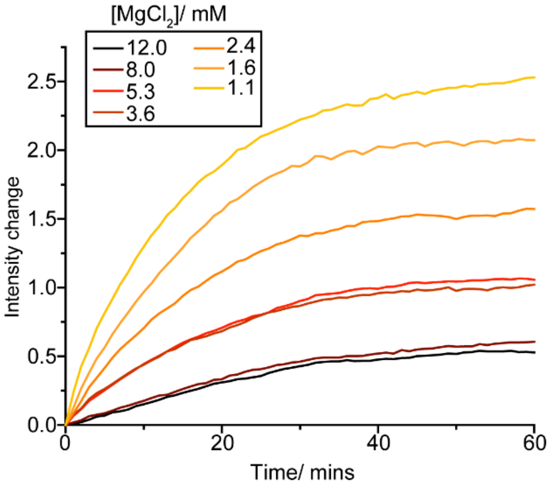

Figure S9. Real-time monitoring of the hexokinase catalysed phosphorylation of glucose at different concentrations of $MgCl_2$. Conditions: 1 mM ATP, 10 mM glucose, 8 μM [**Eu.1**]$^+$, variable $MgCl_2$, 0.5 U mL^{-1} hexokinase, 10 mM HEPES, pH 7.0, λ_{exc} = 292 – 366 nm, λ_{em} = 615 - 625 nm, integration time 60 – 400 μs, measurements taken every 60 s, average of triplicate reactions

Different [NaCl]

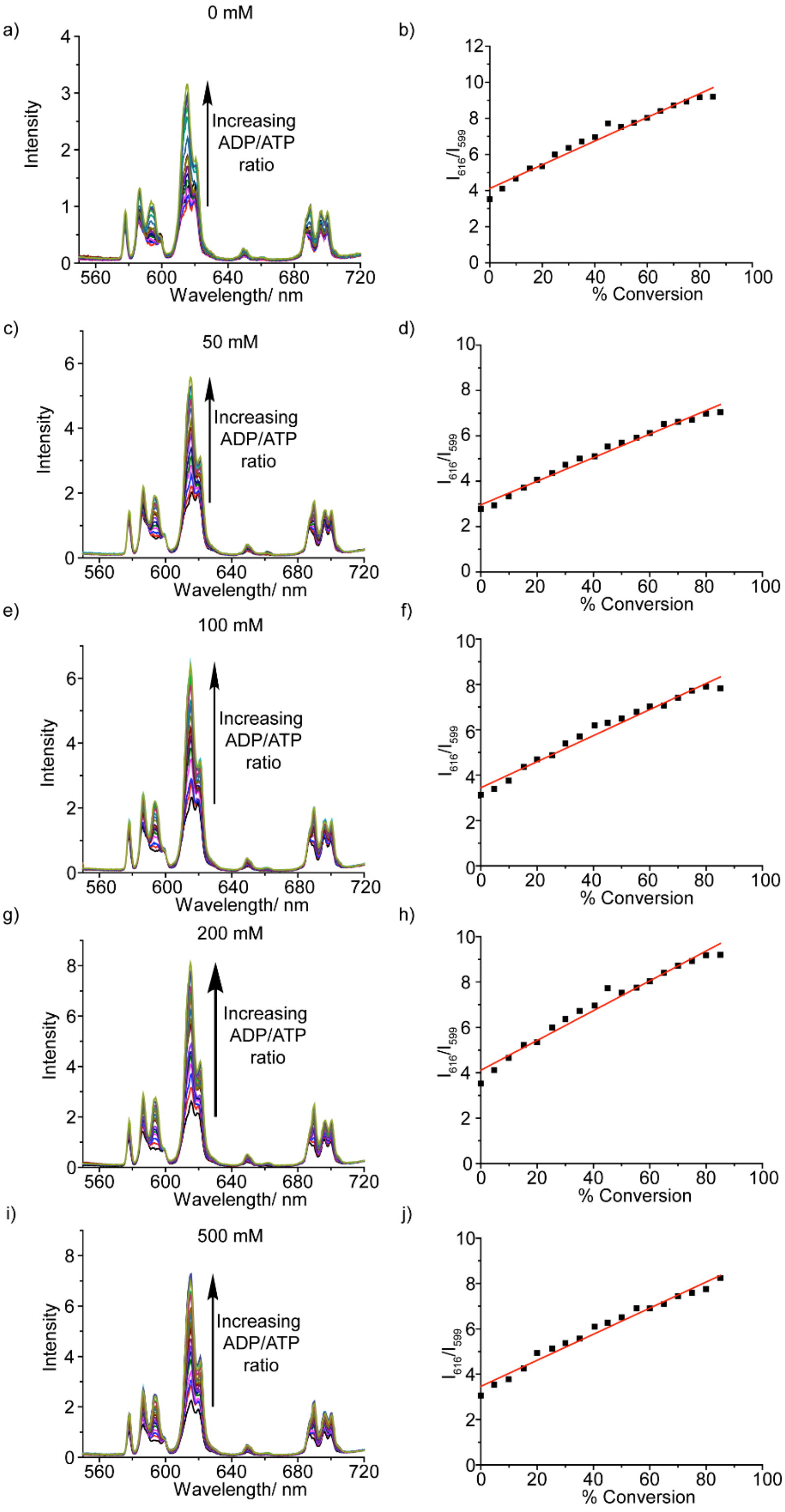

Figure S10. Fluorometer-based kinase simulation at different ionic strengths ([NaCl]): 0 mM (a) and b)), 50 mM (c) and d)), 100 mM (e) and f)), 200 mM (g) and h)) and 500 mM (i) and j)) NaCl. a), c), e), g) and i) Emission spectra of [**Eu.1**]$^+$ on increasing ADP/ATP ratio, b), d), f), h) and j) Plot of emission intensity ratio 616/599 nm versus percentage conversion of ATP to ADP. 8 μM [**Eu.1**]$^+$, 1 mM ATP+ADP, 5 mM $MgCl_2$, 0 – 500 mM NaCl, 10 mM HEPES, pH 7.0, λ_{exc} = 330 nm

Different buffer concentrations

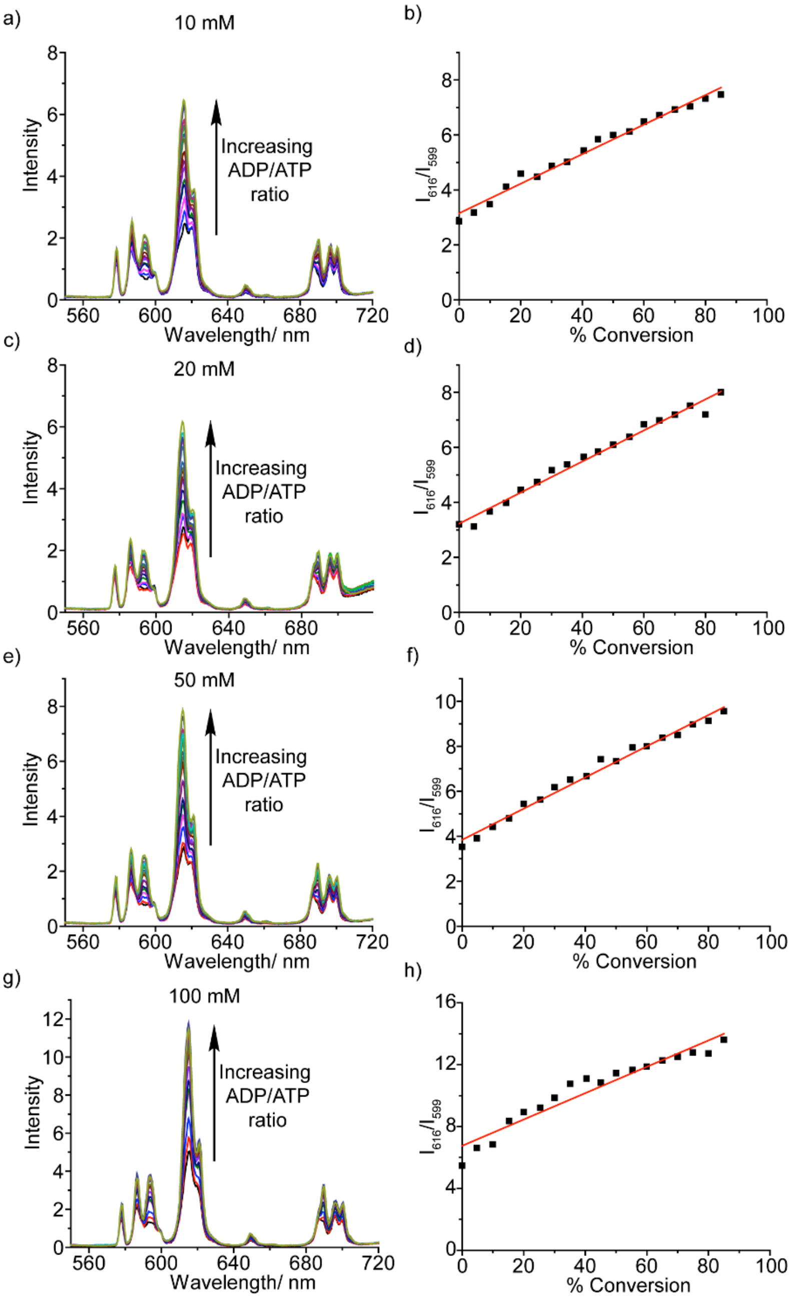

Figure S11. Fluorometer-based kinase simulation at different [Tris]: 10 mM (a) and b)), 20 mM (c) and d)), 50 mM (e) and f)), and 100 mM (g) and h)) Tris, pH 7.5. a), c), e), and g) Emission spectra of [**Eu.1**]$^+$ on increasing ADP/ATP ratio, b), d), f), and h) Plot of emission intensity ratio 616/599 nm versus percentage conversion of ATP to ADP. 8 μM [**Eu.1**]$^+$, 1 mM [ATP+ADP], 5 mM $MgCl_2$, 10 – 100 mM Tris, pH 7.5, λ_{exc} = 330 nm

Hexokinase different substrates

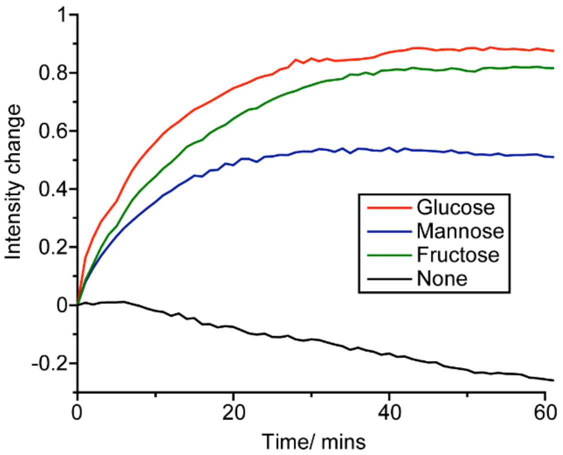

Figure S12. Real-time monitoring of the hexokinase catalysed phosphorylation of glucose, mannose and fructose. Conditions: 1 mM ATP, 10 mM sugar, 8 µM [**Eu.1**]$^+$, 5 mM $MgCl_2$, 10 mM HEPES, pH 7.0, λ_{exc} = 292 – 366 nm, λ_{em} = 615 - 625 nm, integration time 60 – 400 µs, measurements taken every 60 s, average of triplicate reactions

Concentration of [Eu.1]$^+$

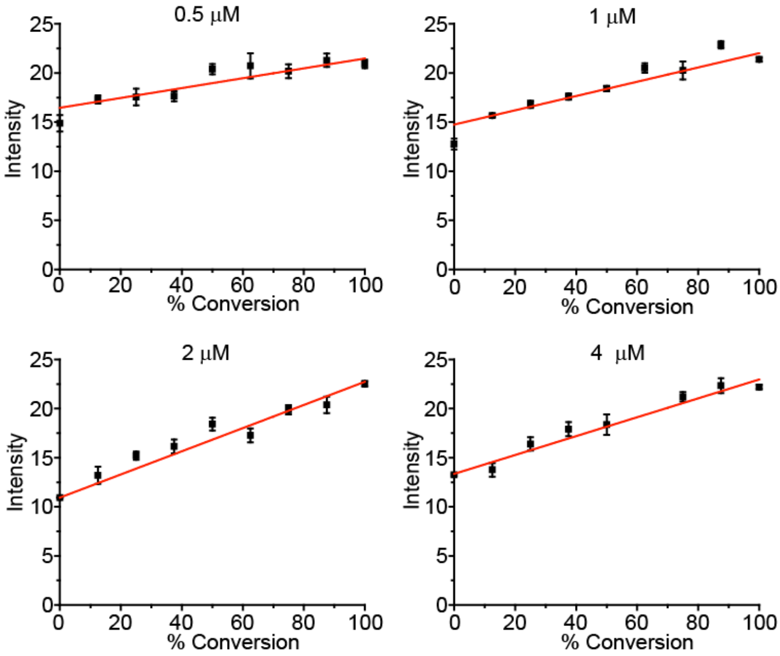

Figure S13. Plate-based kinase simulation using time-resolved method using different concentrations of [**Eu.1**]$^+$: 0.5 µM (a), 1 µM (b), 2 µM (c), 4 µM (d). 1 mM [ATP+ADP], 5 mM $MgCl_2$, 10 mM HEPES, pH 7.0, λ_{exc} = 292 – 366 nm, λ_{em} = 615 - 625 nm, integration time 60 – 400 µs

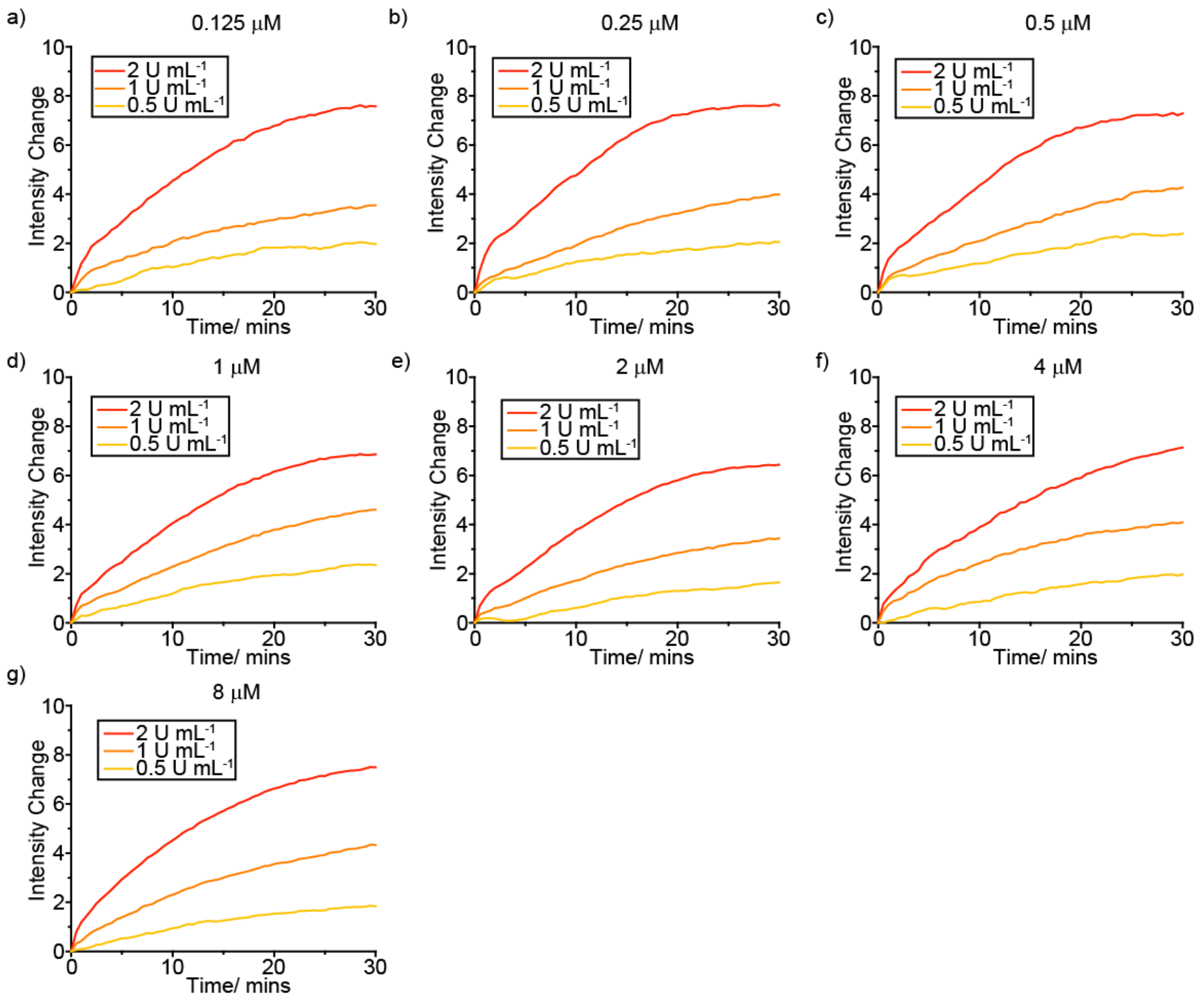

Figure S14. Real-time monitoring of the hexokinase catalysed phosphorylation of glucose at three enzyme concentrations (2, 1, 0.5 and 0 U mL^{-1}) using different concentrations of [**Eu.1**]$^+$: a) 0.125, b) 0.25, c) 0.5, d) 1, e) 2, f) 4 and g) 8 μM. Conditions: 1 mM ATP, 10 mM glucose, variable [**Eu.1**]$^+$, 5 mM $MgCl_2$, 10 mM HEPES, pH 7.0, λ_{exc} = 292 – 366 nm, λ_{em} = 615 - 625 nm, integration time 60 – 400 μs, measurements taken every 30 s, average of triplicate reactions

Different pH

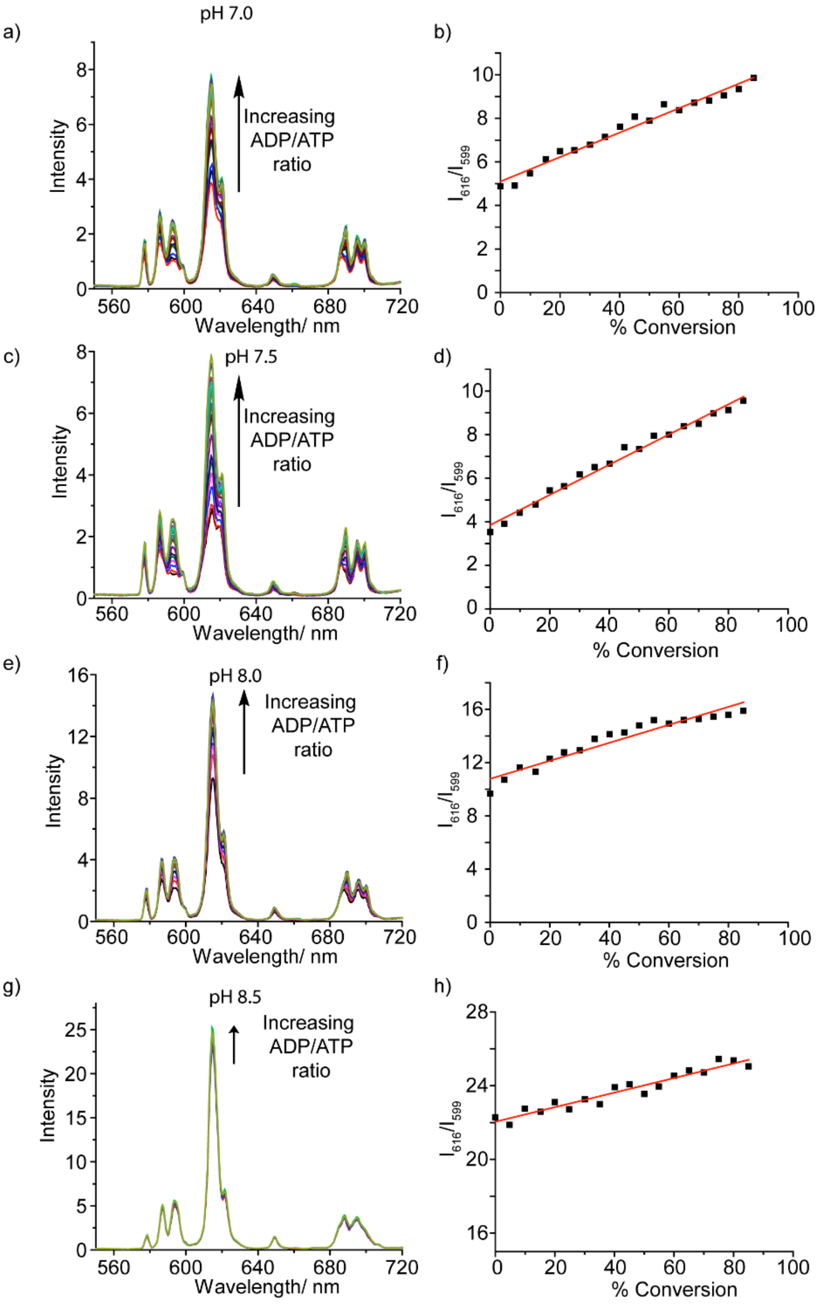

Figure S15. Fluorometer-based kinase simulation at different pHs: pH 7.0 (a) and b)), pH 7.5 (c) and d)), pH 8.0 (e) and f)) and pH 8.5 (g). a), c), e), and g) Emission spectra of [**Eu.1**]$^+$ on increasing ADP/ATP ratio, b), d), f) and h) Plot of emission intensity ratio 616/599 nm versus percentage conversion of ATP to ADP. 8 μM [**Eu.1**]$^+$, 1 mM [ATP+ADP], 5 mM $MgCl_2$, 50 mM Tris, variable pH, λ_{exc} = 330 nm

Additives

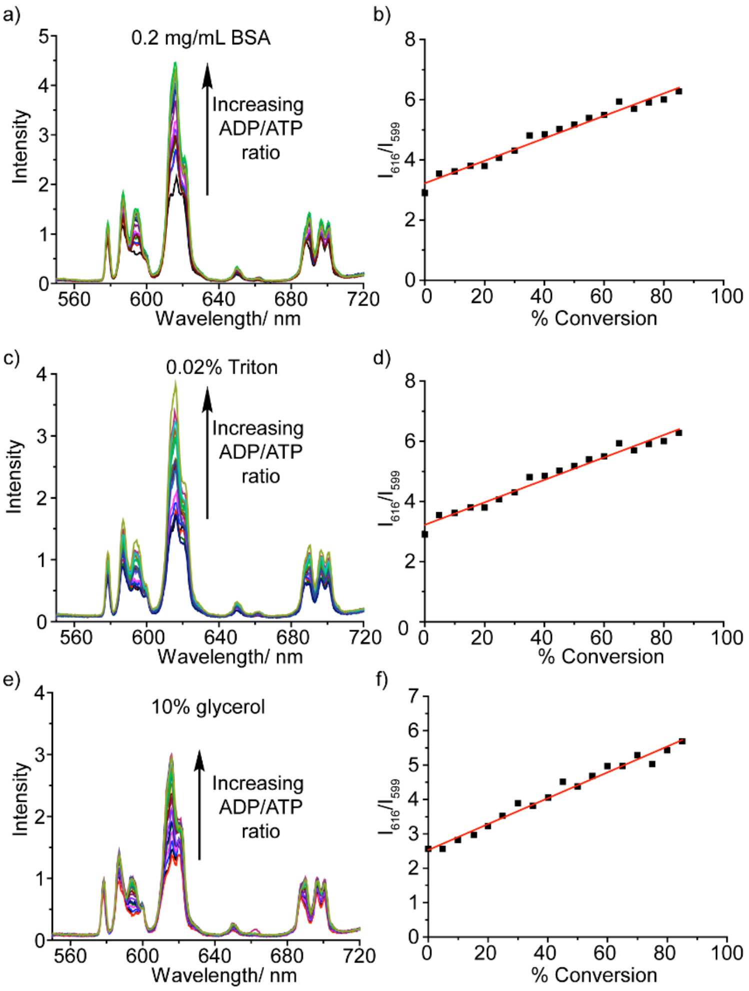

Figure S16. Fluorometer-based kinase simulation with different additives: 0.2 mg/mL BSA (a) and b)), 0.02% Triton X-100 (c) and d)), and 10% glycerol (e) and f)). a), c), and e) Emission spectra of [**Eu.1**]$^+$ on increasing ADP/ATP ratio, b), d), and f) of emission intensity ratio 616/599 nm versus percentage conversion of ATP to ADP. 8 μM [**Eu.1**]$^+$, 1 mM [ATP+ADP], 5 mM $MgCl_2$, 10 mM HEPES, pH 7.0, λ_{exc} = 330 nm

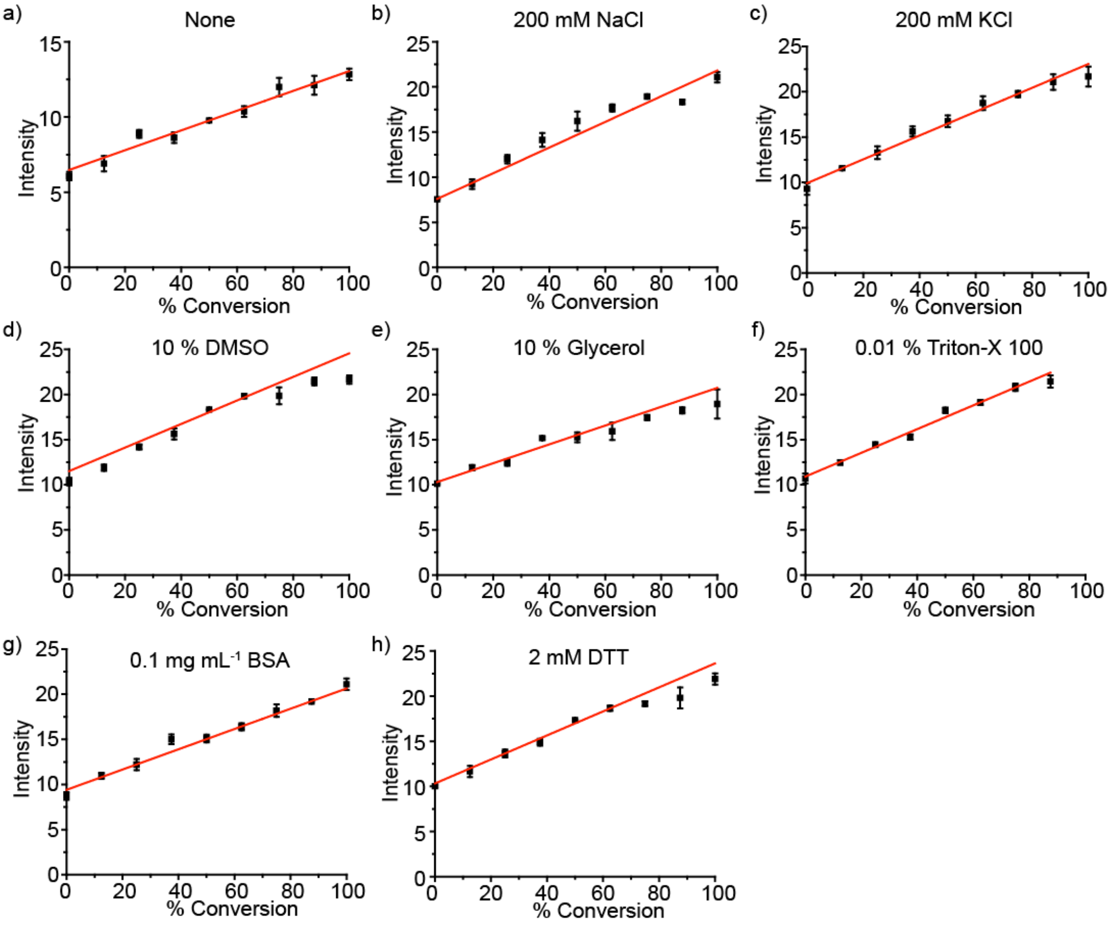

Figure S17. Plate-based kinase simulation using time-resolved method with different buffer additives: None (a), 200 mM NaCl (b), 200 mM KCl (c), 10 % DMSO (d), 10 % glycerol (e), 0.01 % Triton-X 100 (f), 0.1 mg mL^{-1} BSA (g), and 2 mM DTT (h). 8 μM [**Eu.1**]$^+$, 1 mM [ATP+ADP], 5 mM $MgCl_2$, 10 mM HEPES, pH 7.0, λ_{exc} = 292 – 366 nm, λ_{em} = 615 - 625 nm, integration time 60 – 400 μs

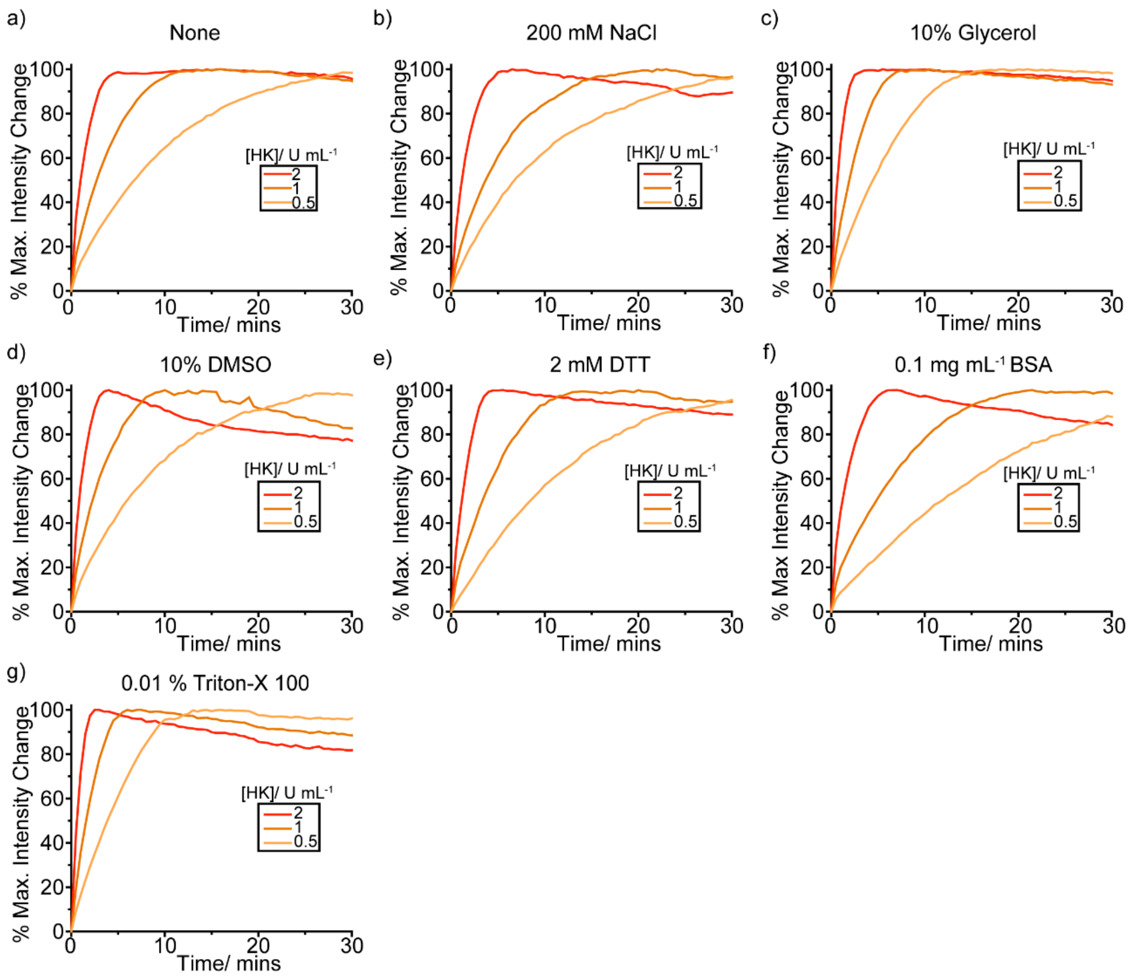

Figure S18. Real-time monitoring of the hexokinase catalysed phosphorylation of glucose at three enzyme concentrations (2, 1 and 0.5 U mL^{-1}) in the presence of various additives: a) none, b) 200 mM NaCl, c) 10% Glycerol, d) 10% DMSO, e) 2 mM DTT, f) 0.1 mg mL^{-1} BSA and g) 0.01% Triton-X 100. Conditions: 1 mM ATP, 10 mM glucose, 8 μM [**Eu.1**]$^{+}$, 5 mM $MgCl_2$, 10 mM HEPES, pH 7.0, λ_{exc} = 292 – 366 nm, λ_{em} = 615 - 625 nm, integration time 60 – 400 μs, measurements taken every 30 s, average of triplicate reactions

Different M^{2+}

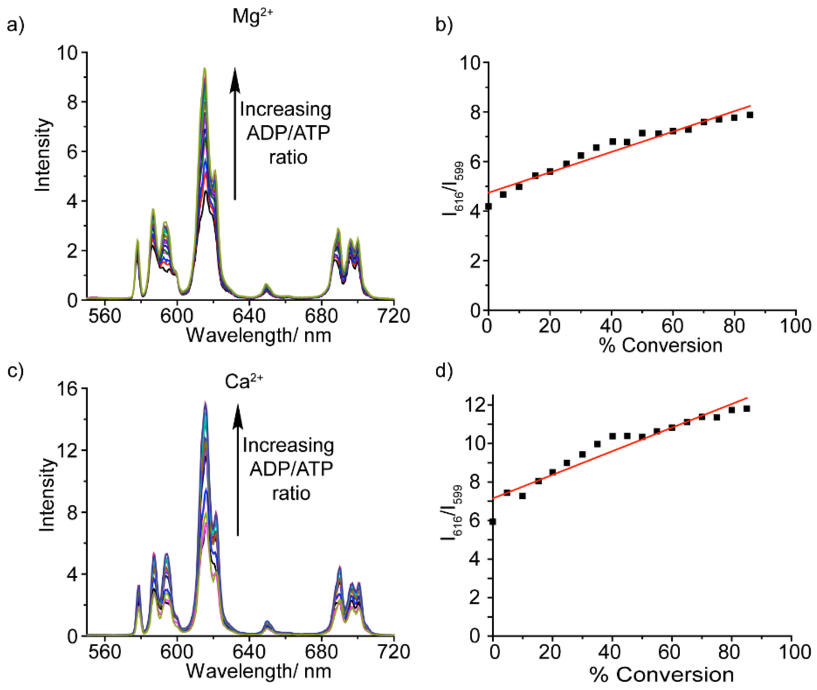

Figure S19. Fluorometer-based kinase simulation with different MCl_2: $MgCl_2$ (a) and b)), and $CaCl_2$ (c) and d)). a) and c) Emission spectra of [**Eu.1**]$^+$ on increasing ADP/ATP ratio, b) and d) of emission intensity ratio 616/599 nm versus percentage conversion of ATP to ADP. 8 μM [**Eu.1**]$^+$, 1 mM [ATP+ADP], 5 mM MCl_2, 10 mM HEPES, pH 7.0, λ_{exc} = 330 nm

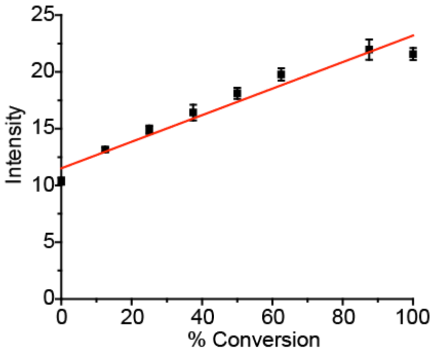

Figure S20. Plate-based kinase simulation using time-resolved method with $CaCl_2$. 8 μM [**Eu.1**]$^+$, 1 mM [ATP+ADP], 5 mM $CaCl_2$, 10 mM HEPES, pH 7.0, λ_{exc} = 330 nm, λ_{em} = 620 nm, integration time 60 – 400 μs

Temperature

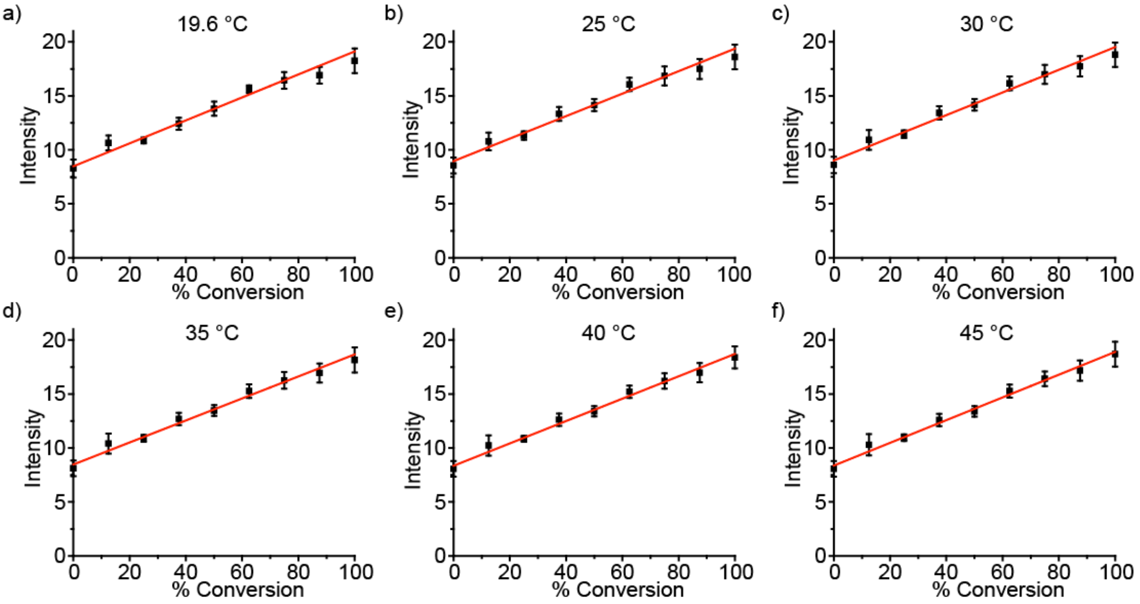

Figure S21. Plate-based kinase simulation using time-resolved method at different temperatures: 19.6 °C (a), 25 °C (b), 30 °C (c), 35 °C (d), 40 °C (e), and 45 °C (f). 8 μM [**Eu.1**]+, 1 mM [ATP+ADP], 5 mM $MgCl_2$, 10 mM HEPES, pH 7.0, λ_{exc} = 292 – 366 nm, λ_{em} = 615 - 625 nm, integration time 60 – 400 μs

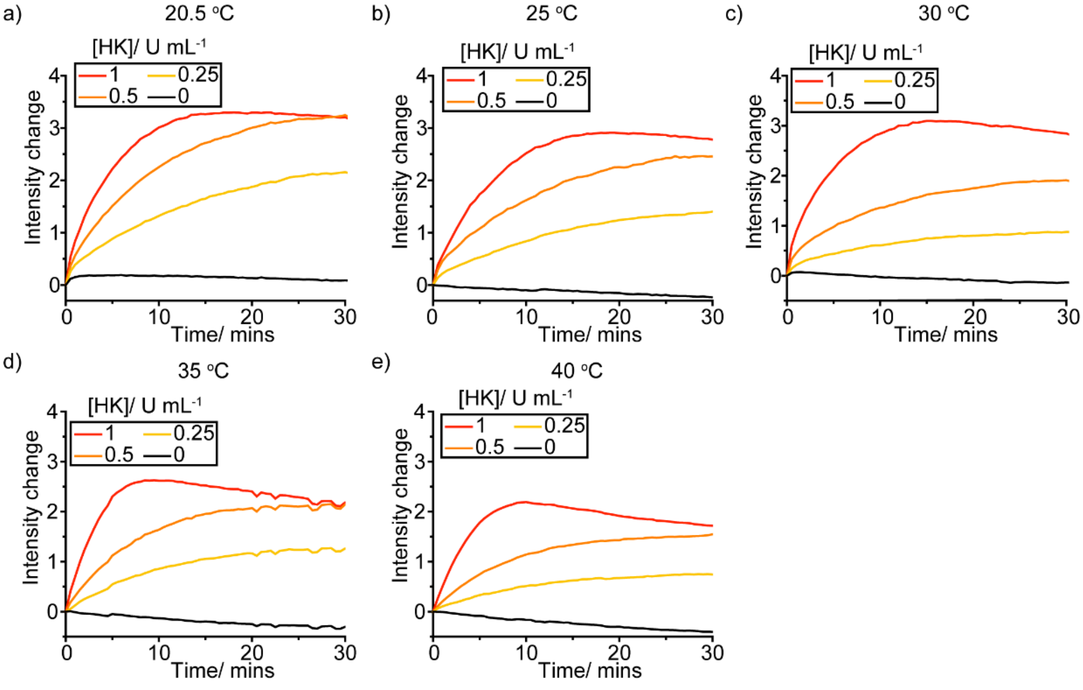

Figure S22. Real-time monitoring of the hexokinase catalysed phosphorylation of glucose at three enzyme concentrations (2, 1, 0.5 and 0 U mL-1) at different temperatures: a) 20.5 °C, b) 25 °C, c) 30 °C, d) 35 °C and, e) 40 °C. Conditions: 1 mM ATP, 10 mM glucose, 8 μM [**Eu.1**]+, 5 mM $MgCl_2$, 10 mM HEPES, pH 7.0, λ_{exc} = 292 – 366 nm, λ_{em} = 615 - 625 nm, integration time 60 – 400 μs, measurements taken every 30 s, average of triplicate reactions

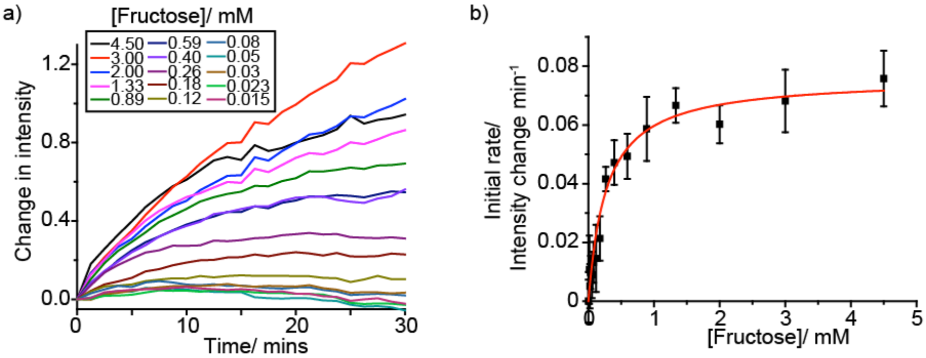

Figure S23. Real-time monitoring of the hexokinase catalysed phosphorylation of fructose at different concentrations of fructose. A) Change in intensity against time at different fructose concentrations, b) Fit of initial rate against fructose concentration to a Michaelis-Menten equation Conditions: 1 mM ATP, 8 μM [**Eu.1**]$^{+}$, 5 mM $MgCl_2$, 0.25 U mL^{-1} hexokinase, 10 mM HEPES, pH 7.0, λ_{exc} = 292 – 366 nm, λ_{em} = 615 - 625 nm, integration time 60 – 400 μs, measurements taken every 75 s, average of triplicate reactions

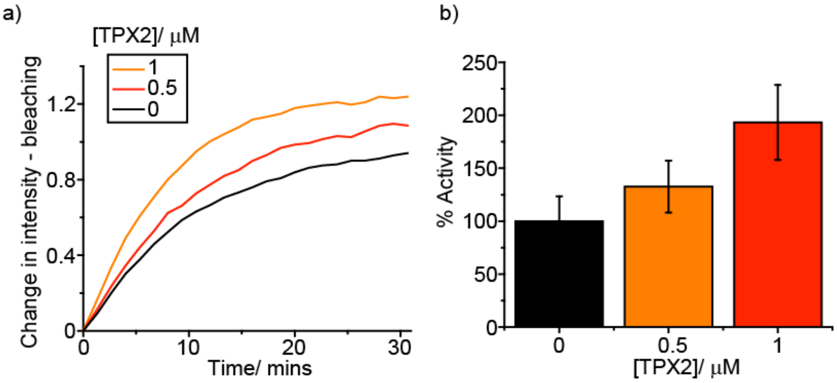

Figure S24. Real-time monitoring of the activation of AurA by TPX2 (a), showing an increase initial reaction rate (b), shown as a % activity with no TPX2. Conditions: 1 mM ATP, 1 μM AurA, 0.5 mM kemptide, 0.25 mM DTT, 5 mM $MgCl_2$, 8 μM **[Eu.1]**$^{+}$, 2.5% glycerol, 50 mM NaCl, 10 mM HEPES, pH 7.0, λ_{exc} = 292 – 366 nm, λ_{em} = 615 - 625 nm, integration time = 60 – 400 μs

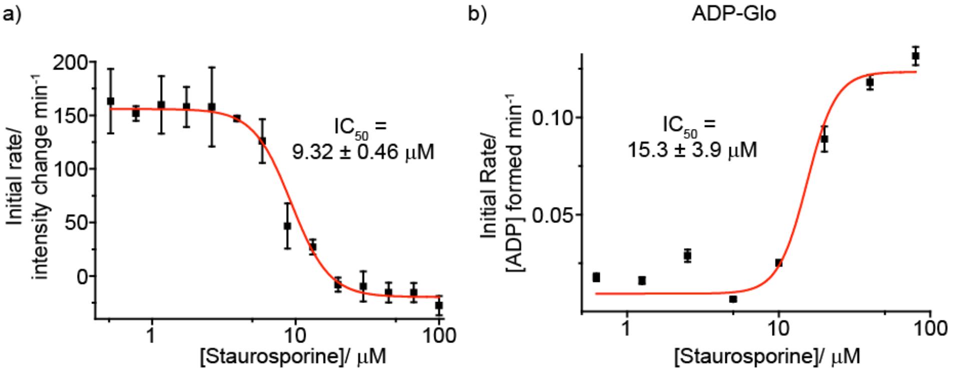

Figure S25. Comparison of staurosporine inhibition IC50, using **[Eu.1]**$^{+}$ in real-time (a) and ADP-Glo (b) Conditions: 1 mM ATP, 50 nM AurA, 0.5 mM kemptide, 0.25 mM DTT, 5 mM $MgCl_2$, 8 μM **[Eu.1]**$^{+}$, 2.5% glycerol, 50 mM NaCl, 5% DMSO, 10 mM HEPES, pH 7.0, for **[Eu.1]**$^{+}$: λ_{exc} = 292 – 366 nm, λ_{em} = 615 - 625 nm, integration time = 60 – 400 μs

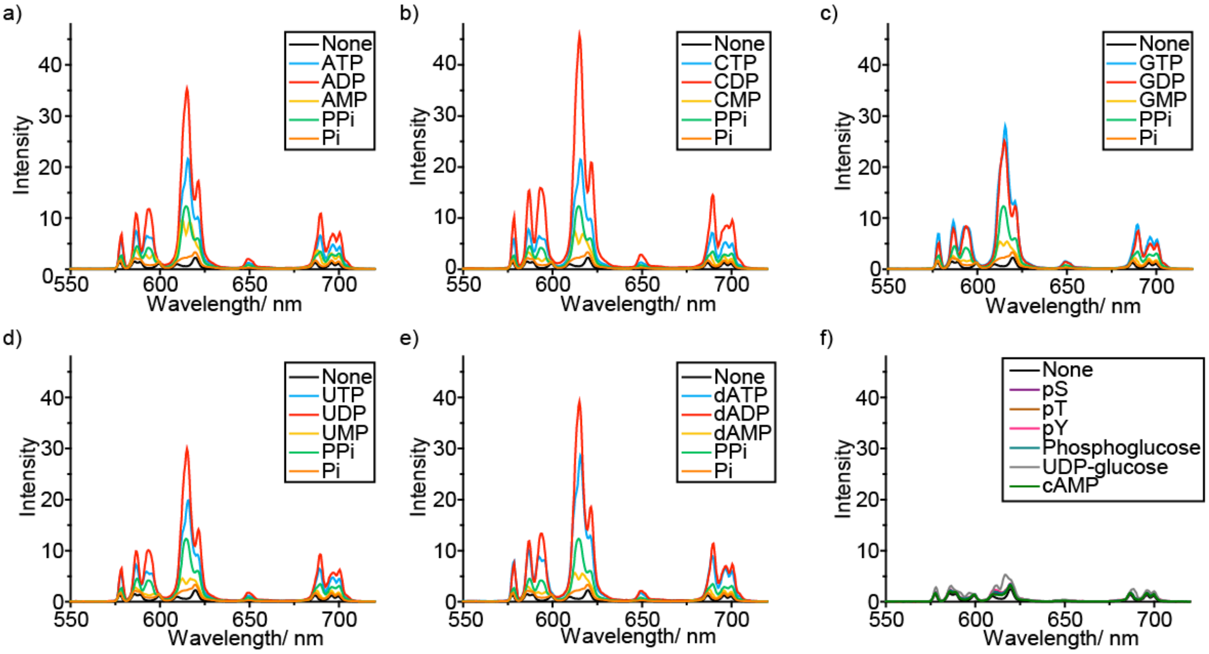

Figure S26. Effect of the addition of various phosphoanions (1 mM) on the emission spectra of [**Eu.1**]+. Conditions: 8 μM [**Eu.1**]+, 1 mM phosphoanion, 10 mM HEPES, pH 7.0, λ_{exc} = 330 nm

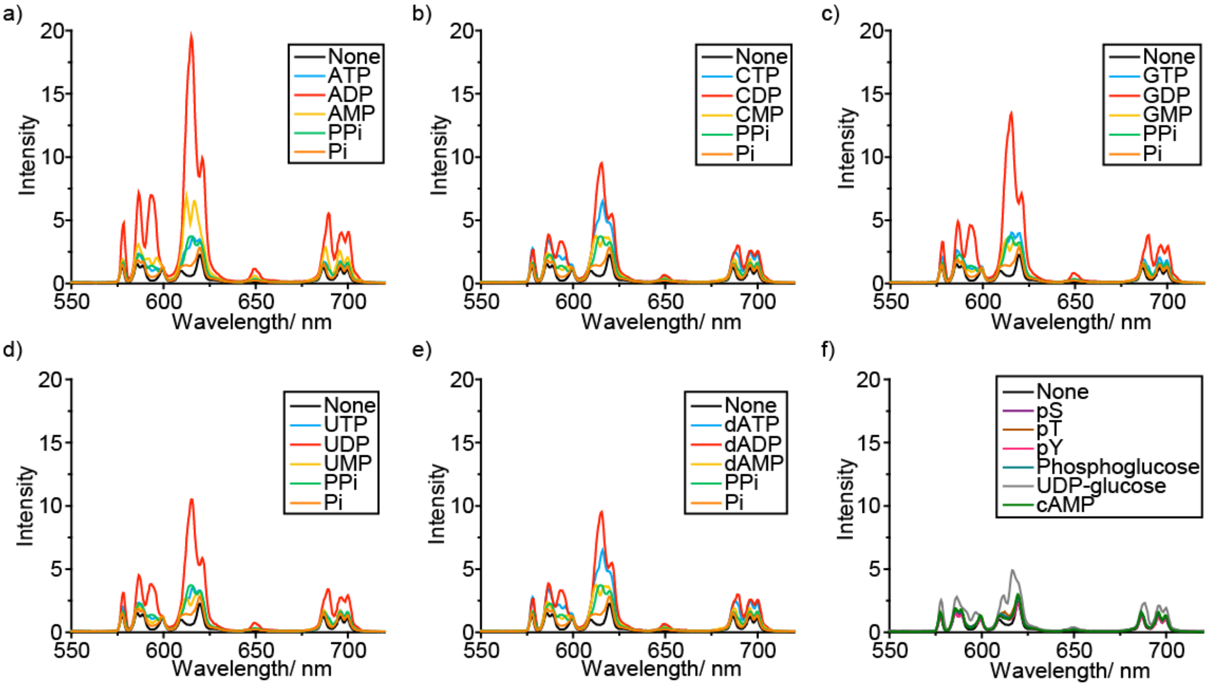

Figure S27. Effect of the addition of various phosphoanions (1 mM) on the emission spectra of [**Eu.1**]+ in the prescence of 5 mM $MgCl_2$. Conditions: 8 μM [**Eu.1**]+, 1 mM phosphoanion, 5 mM $MgCl_2$, 10 mM HEPES, pH 7.0, λ_{exc} = 330 nm

Other enzyme reactions

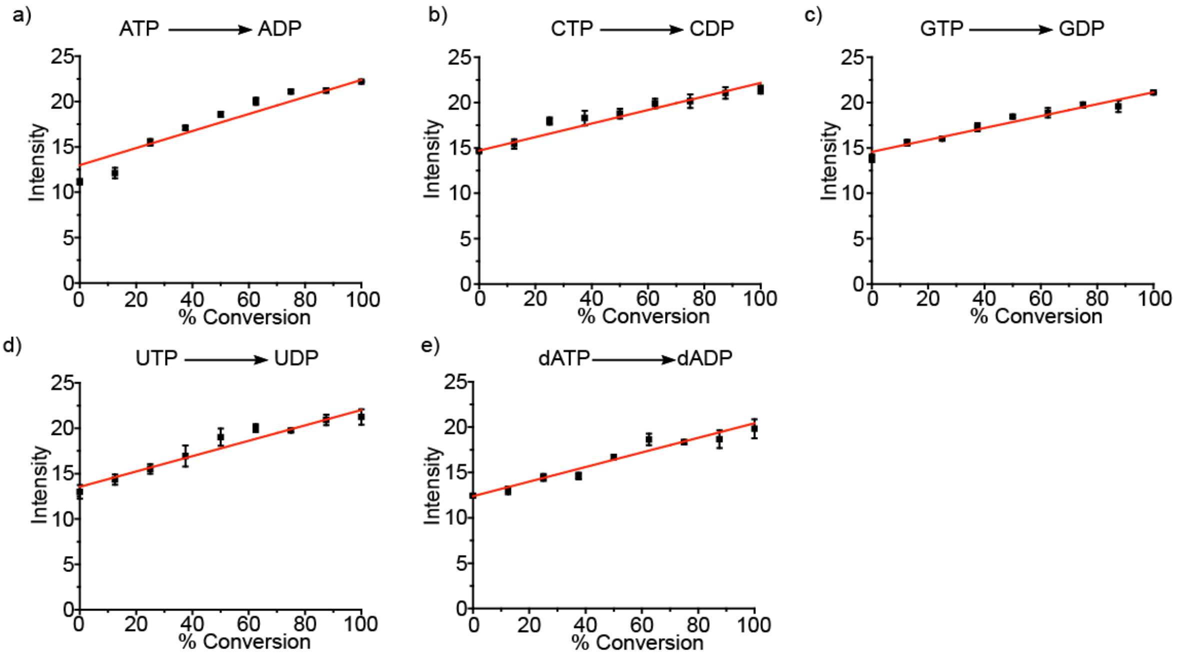

Figure S28. Plate-based enzyme simulations of NTP to NDP enzyme reactions. a) ATP to ADP, b) CTP to CDP, c) GTP to GDP, d) UTP to UDP, and e) dATP to dADP. 8 μM [**Eu.1**]$^+$, 1 mM [NTP + NDP], 5 mM $MgCl_2$, 10 mM HEPES, pH 7.0, λ_{exc} = 292 – 366 nm, λ_{em} = 615 - 625 nm, integration time 60 – 400 μs

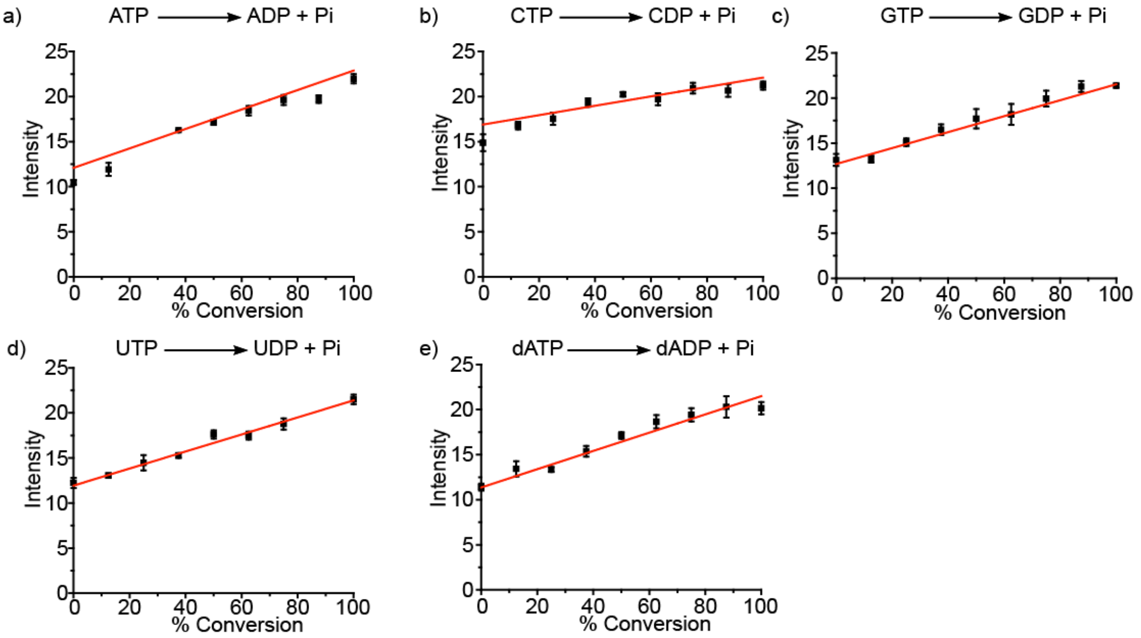

Figure S29. Plate-based NTPase simulations of NTP to NDP + Pi. a) ATP to ADP + Pi, b) CTP to CDP + Pi, c) GTP to GDP + Pi, d) UTP to UDP + Pi and e) dATP to dADP + Pi. 8 μM [**Eu.1**]$^+$, 1 mM [NTP + (NDP + Pi)], 5 mM $MgCl_2$, 10 mM HEPES, pH 7.0, λ_{exc} = 292 – 366 nm, λ_{em} = 615 - 625 nm, integration time 60 – 400 μs

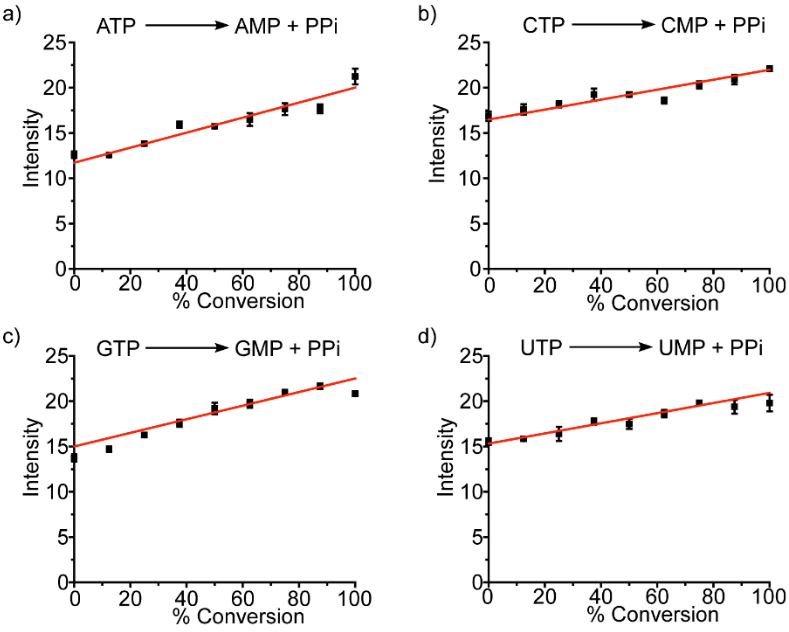

Figure S30. Plate-based nucleoside triphosphate pyrophosphohydrolase simulation of NTP to NMP + PPi. a) ATP to AMP + PPi, b) CTP to CMP + PPi, c) GTP to GMP + PPi and d) UTP to UMP + PPi. 8 μM [**Eu.1**]+, 1 mM [NTP + (NMP + PPi)], 5 mM $MgCl_2$, 10 mM HEPES, pH 7.0, λ_{exc} = 292 – 366 nm, λ_{em} = 615 - 625 nm, integration time 60 – 400 μs

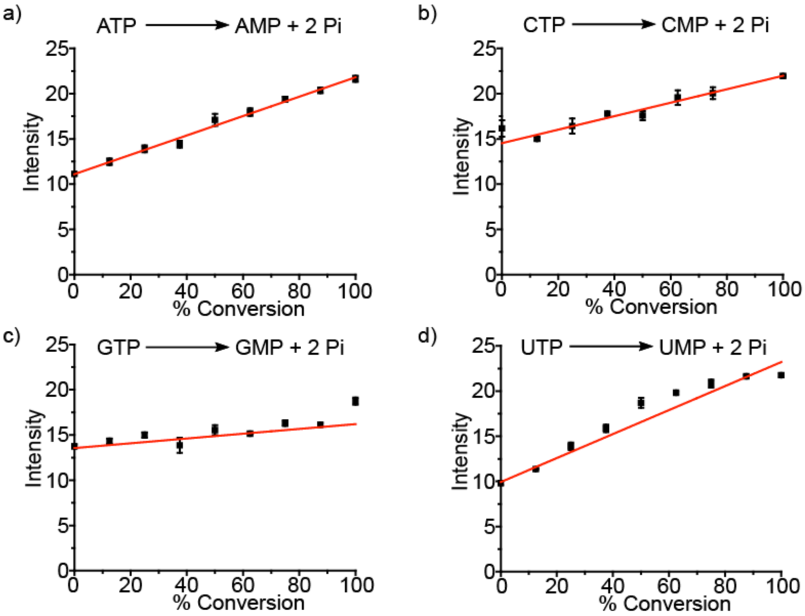

Figure S31. Plate-based enzyme simulations of NTP to NMP + 2 Pi. a) ATP to AMP + 2 Pi, b) CTP to CMP + 2 Pi, c) GTP to GMP + 2 Pi and d) UTP to UMP + 2 Pi. 8 μM [**Eu.1**]+, 1 mM [NTP + (NMP + 2 Pi)], 5 mM $MgCl_2$, 10 mM HEPES, pH 7.0, λ_{exc} = 292 – 366 nm, λ_{em} = 615 - 625 nm, integration time 60 – 400 μs

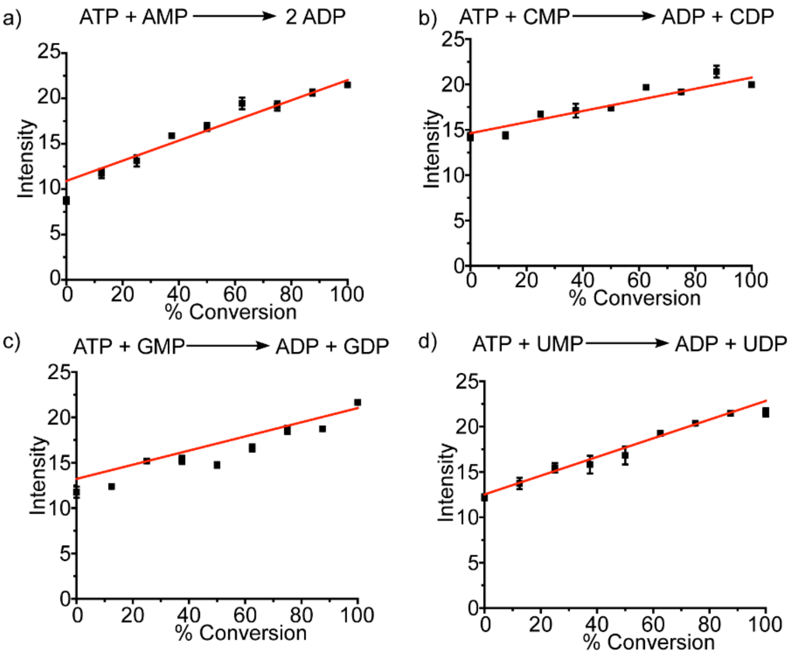

Figure S32. Plate-based enzyme simulations of ATP + NMP to ADP + NDP. a) ATP + AMP to ADP + NDP, b) ATP + CMP to ADP + CDP, c) ATP + GMP to ADP + GDP and d) ATP + UMP + ADP + UDP. 8 μM [**Eu.1**]$^+$, 1 mM [(ATP + NMP) + (ADP + NMP)], 5 mM $MgCl_2$, 10 mM HEPES, pH 7.0, λ_{exc} = 292 – 366 nm, λ_{em} = 615 - 625 nm, integration time 60 – 400 μs

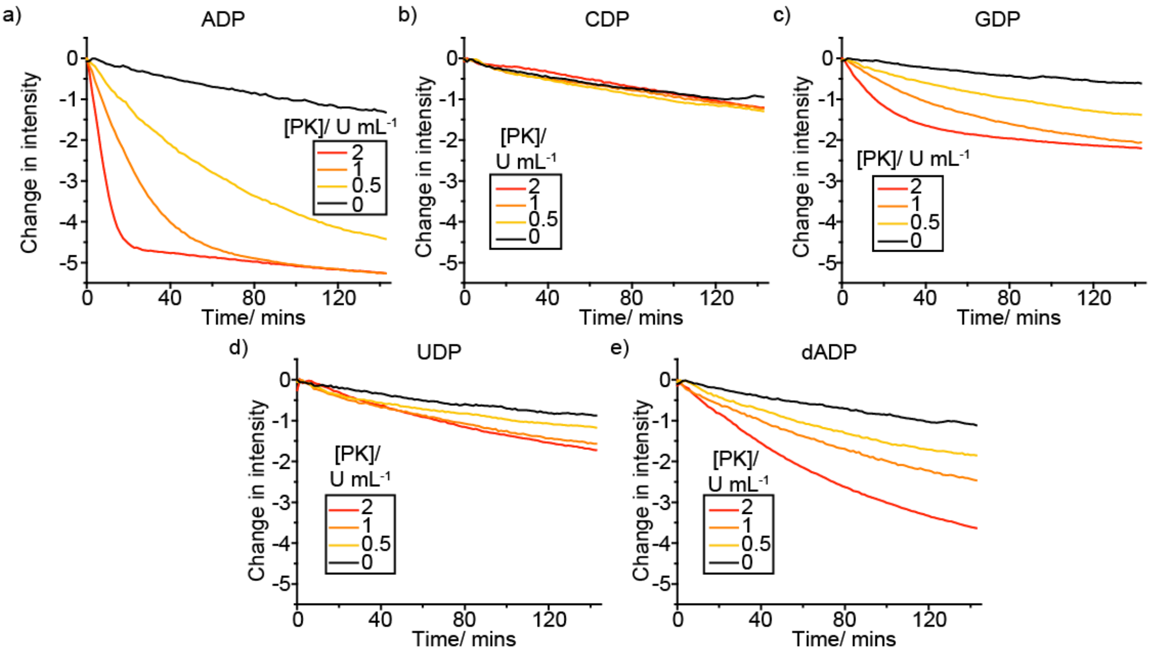

Figure S33. Real-time monitoring of the pyruvate kinase catalysed conversion of phospho(enol) pyruvate to pyruvate, using ADP (a), CDP (b), GDP (c), UDP (d) and dADP (e) at three enzyme concentrations (2, 1, 0.5 and 0 U mL^{-1}). Conditions: 1 mM NDP, 1 mM phosphor(enol) pyruvate, 8 μM [**Eu.1**]$^+$, 5 mM $MgCl_2$, 50 mM KCl, 10 mM HEPES, pH 7.0, λ_{exc} = 292 – 366 nm, λ_{em} = 615 - 625 nm, integration time 60 – 400 μs, measurements taken every 80 s, average of triplicate reactions

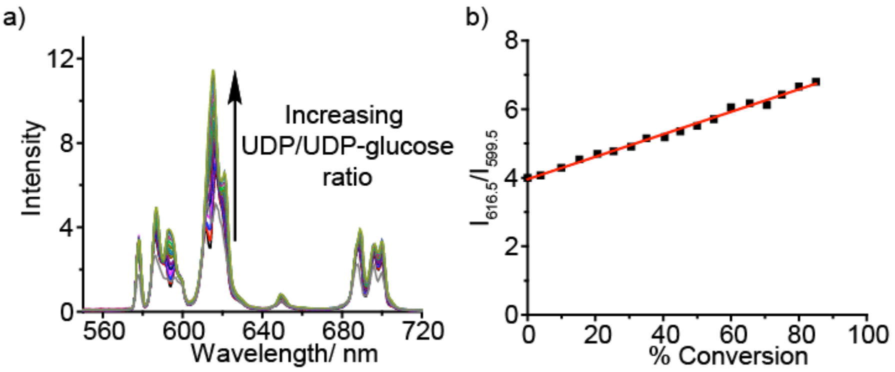

Figure S34. Fluorometer-based glycosyl transferase simulation: UDP-glucose + glucose to UDP + maltose. a) Emission spectra of [**Eu.1**]$^+$ on increasing UDP/UDP-glucose ratio, b) Plot of emission intensity ratio 616.5/599.5 nm versus percentage conversion UDP-glucose to UDP. 8 μM [**Eu.1**]$^+$, 1 mM [UDP-glucose + UDP], 9 mM glucose, 1 mM [glucose + maltose], 2 mM $MgCl_2$, 10 mM HEPES, pH 7.0, λ_{exc} = 330 nm

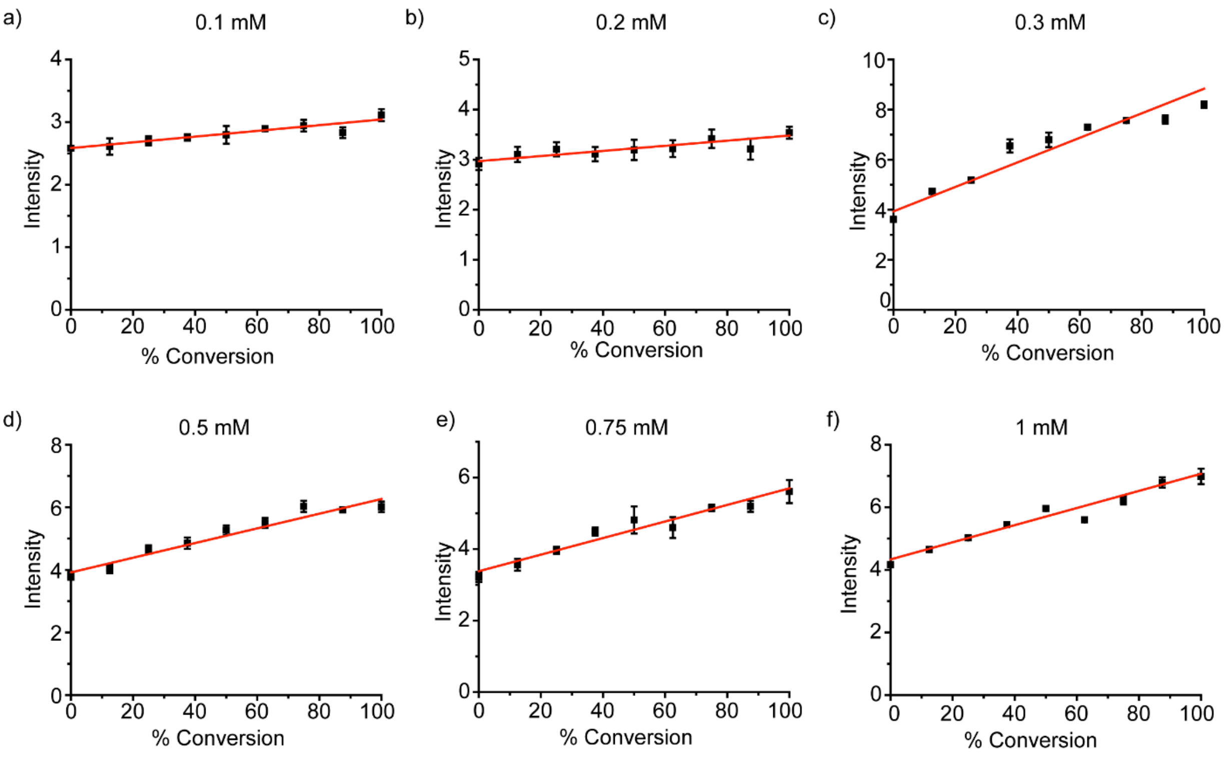

Figure S35. Plate-based glycosyl transferase simulation (UDP-glucose + glucose to UDP + maltose) using time-resolved method with different UDP-glucose and UDP concentrations: 0.1 (a), 0.2 (b), 0.3 (c), 0.5 (d), 0.75 (e), and 1.0 (f) mM UDP-glucose + UDP. 8 μM [**Eu.1**]$^+$, 10 mM [glucose + maltose], 2 mM $MgCl_2$, 10 mM HEPES, pH 7.0, λ_{exc} = 292 – 366 nm, λ_{em} = 615 - 625 nm, integration time 60 – 400 μs

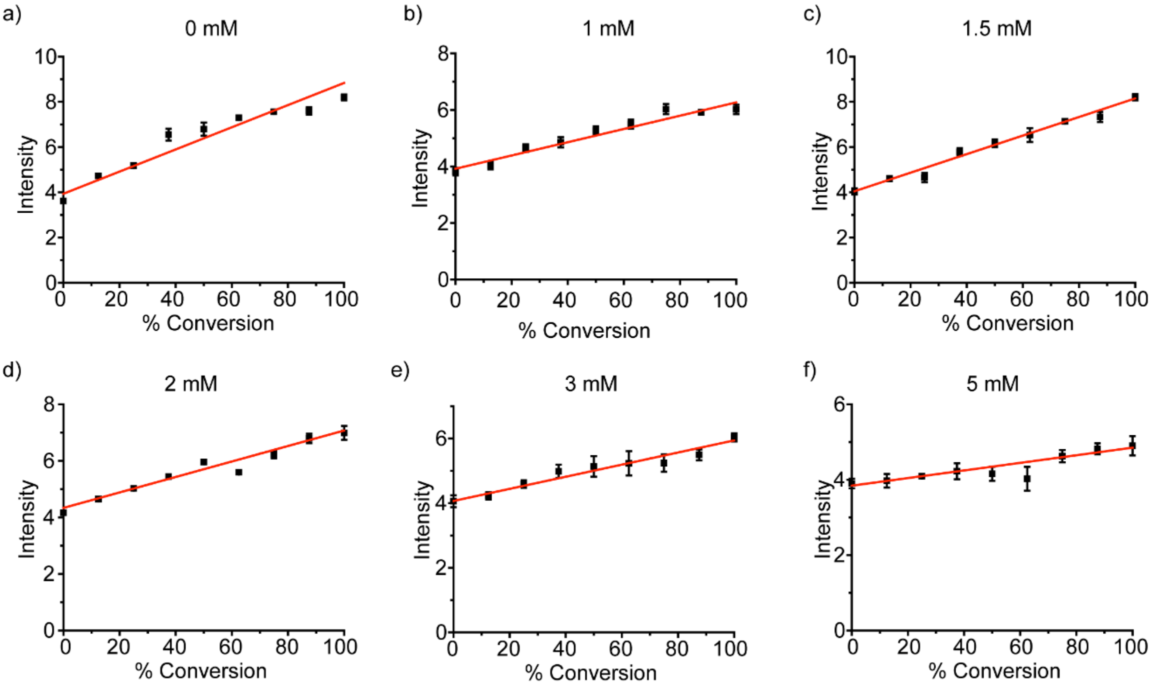

Figure S36. Plate-based glycosyl transferase simulation (UDP-glucose + glucose to UDP + maltose) using time-resolved method with different $MgCl_2$ concentrations: 0 (a), 1 (b), 1.5 (c), 2 (d), 3 (e), and 5 (f) mM $MgCl_2$. 8 μM [**Eu.1**]$^+$, 1 mM [UDP-glucose + UDP], 10 mM [glucose + maltose], 10 mM HEPES, pH 7.0, λ_{exc} = 292 – 366 nm, λ_{em} = 615 - 625 nm, integration time 60 – 400 μs

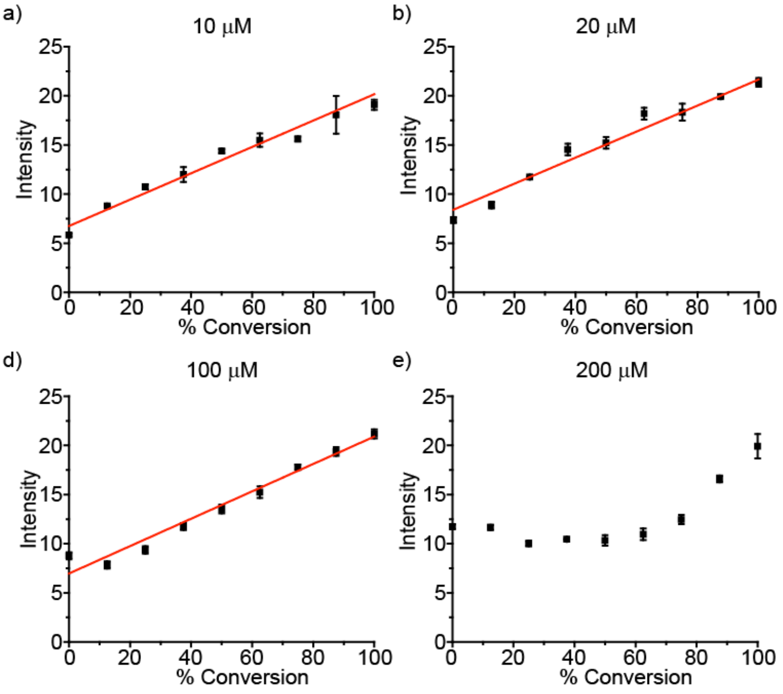

Figure S37. Plate-based glycosyl transferase simulation (UDP-glucose + glucose to UDP + maltose) using time-resolved method with different $MnCl_2$ concentrations: 10 (a), 20 (b), 50 (c), 100 (d) and 200 (e) μM $MnCl_2$. 8 μM [**Eu.1**]$^+$, 1 mM [UDP-glucose + UDP], 10 mM [glucose + maltose], 10 mM HEPES, pH 7.0, λ_{exc} = 292 – 366 nm, λ_{em} = 615 - 625 nm, integration time 60 – 400 μs

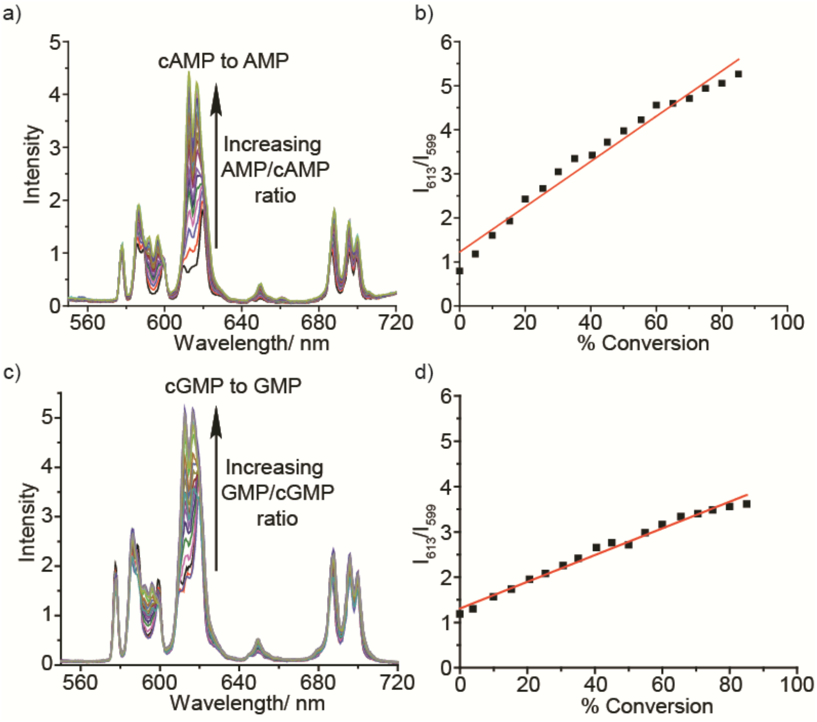

Figure S38. Fluorometer-based phosphodiesterase simulations a) and b) cAMP to AMP, c) and d) cGMP to GMP. a) and c) Emission spectra of [**Eu.1**]$^+$ on increasing AMP/cAMP or GMP/cGMP ratio, b) and d) Plot of emission intensity ratio 613/599 nm versus percentage conversion of cAMP or cGMP to AMP or GMP. 8 μM [**Eu.1**]$^+$, 1 mM [cAMP + AMP or cGMP + GMP], 5 mM $MgCl_2$, 10 mM HEPES, pH 7.0, λ_{exc} = 330 nm

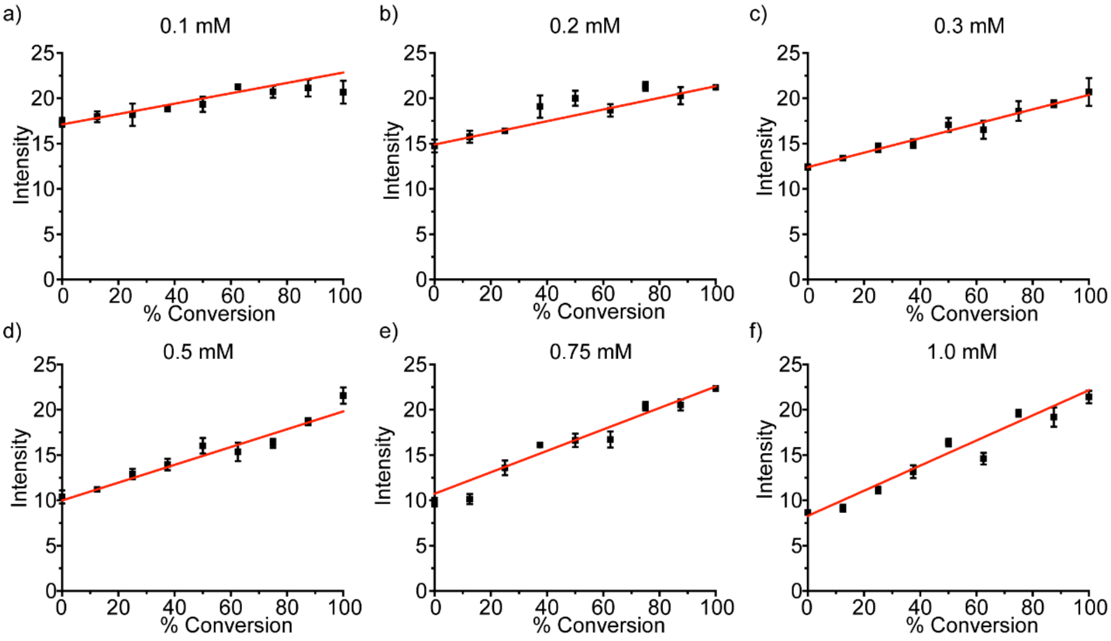

Figure S39. Plate-based cyclic nucleotide phosphodiesterase simulation (cAMP to AMP) using time-resolved method with different anion (cAMP+AMP) concentrations: 0.1 (a), 0.2 (b), 0.3 (c), 0.5 (d), 0.75 (e), and 1.0 (f) mM [cAMP] + [AMP]. 8 μM [**Eu.1**]$^+$, variable [cAMP+AMP], 5 mM $MgCl_2$, 10 mM HEPES, pH 7.0, λ_{exc} = 292 – 366 nm, λ_{em} = 615 - 625 nm, integration time 60 – 400 μs

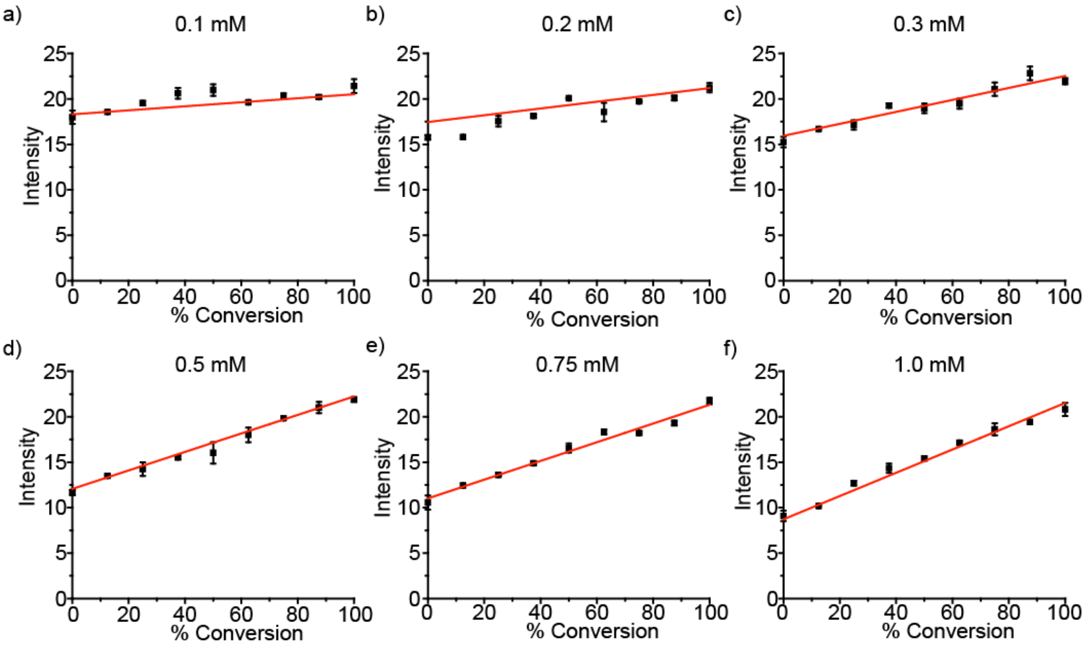

Figure S40. Plate-based cyclic nucleotide phosphodiesterase simulation (cGMP to GMP) using time-resolved method with different anion (cGMP+GMP) concentrations: 0.1 (a), 0.2 (b), 0.3 (c), 0.5 (d), 0.75 (e), and 1.0 (f) mM [cGMP] + [GMP]. 8 µM [**Eu.1**]+, variable [cGMP+GMP], 5 mM $MgCl_2$, 10 mM HEPES, pH 7.0, λ_{exc} = 292 – 366 nm, λ_{em} = 615 - 625 nm, integration time 60 – 400 µs

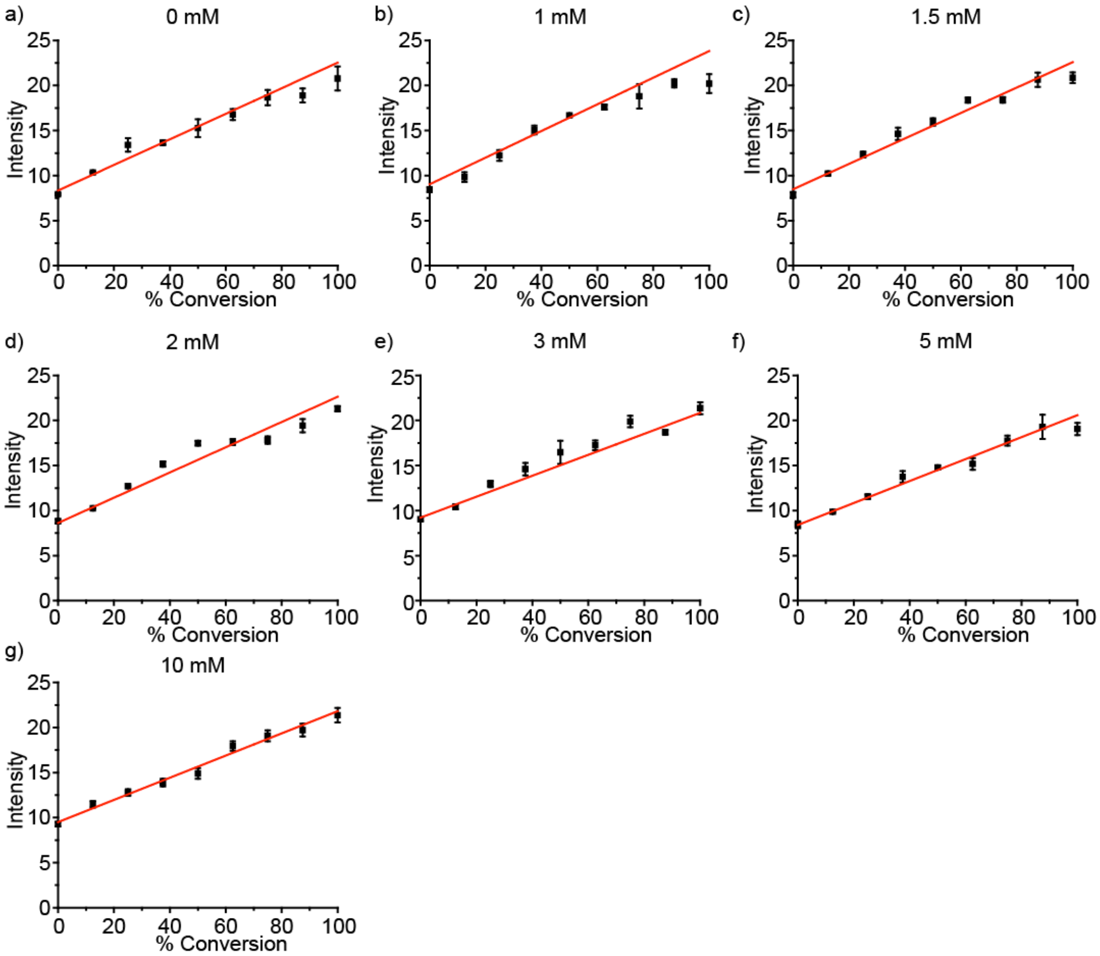

Figure S41. Plate-based cyclic nucleotide phosphodiesterase simulation (cAMP to AMP) using time-resolved method with different $MgCl_2$ concentrations: 0 (a), 1 (b), 1.5 (c), 2 (d), 3 (e), 5 (f), and 10 (g) mM $MgCl_2$. 8 µM [**Eu.1**]$^+$, 1 mM [cAMP+AMP], variable $MgCl_2$, 10 mM HEPES, pH 7.0, λ_{exc} = 292 – 366 nm, λ_{em} = 615 - 625 nm, integration time 60 – 400 µs

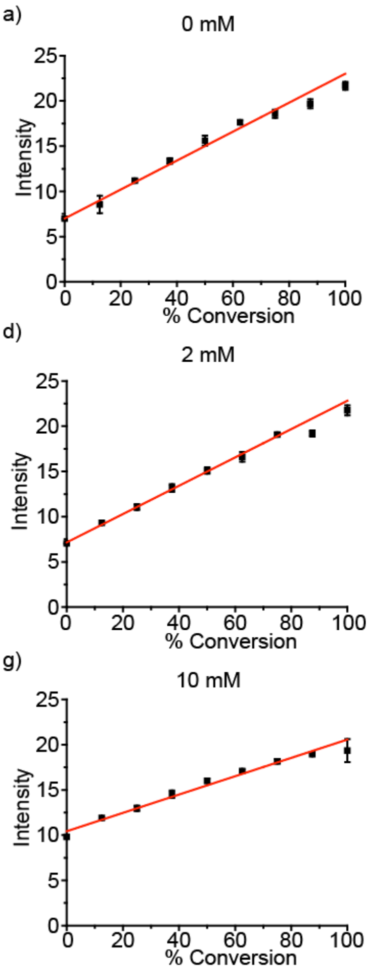

Figure S42. Plate-based cyclic nucleotide phosphodiesterase simulation (cGMP to GMP) using time-resolved method with different $MgCl_2$ concentrations: 0 (a), 1 (b), 1.5 (c), 2 (d), 3 (e), 5 (f), and 10 (g) mM $MgCl_2$. 8 μM [**Eu.1**]$^+$, 1 mM [cGMP+GMP], variable $MgCl_2$, 10 mM HEPES, pH 7.0, λ_{exc} =292 – 366 nm, λ_{em} = 615 - 625 nm, integration time 60 – 400 μs

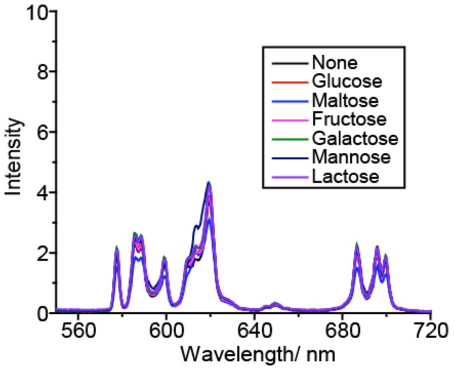

Figure S43. Effect of various sugars (10 mM) on the emission spectra of [**Eu.1**]$^+$ (8 μM) in 10 mM HEPES, pH 7.0, λ_{exc} = 330 nm

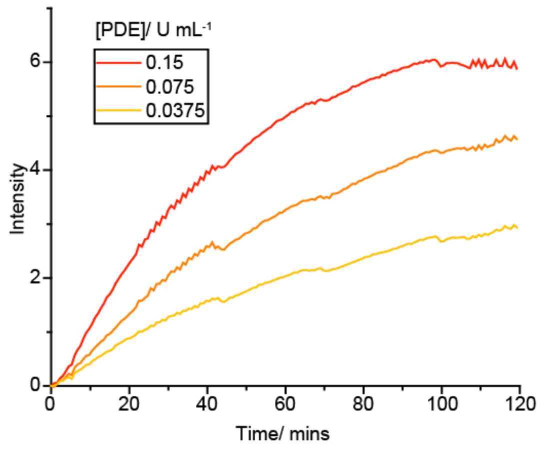

Figure S44. Real-time monitoring of the PDE-catalysed conversion of cAMP to AMP, conditions: 1 mM cAMP, 5 mM $MgCl_2$, 8 μM **[Eu.1]**$^+$, 10 mM HEPES, pH 7.0, λ_{exc} = 292 – 366 nm , λ_{em} = 615 - 625 nm, integration time = 60 – 400 μs

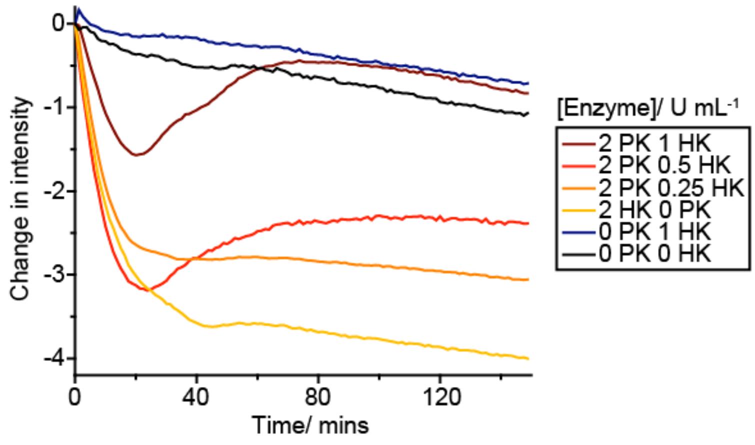

Figure S45. Real-time monitoring of sequential enzyme reactions, involving hexokinase (HK, ATP to ADP) and pyruvate kinase (PK, ADP to ATP). Starting from ADP. Conditions: 1 mM ADP, 1 mM glucose, 1 mM PEP, 5 mM $MgCl_2$, 50 mM KCl, 8 μM **[Eu.1]**$^+$, 10 mM HEPES, pH 7.0, λ_{exc} = 292 – 366 nm, λ_{em} = 615 - 625 nm, integration time = 60 – 400 μs)
